# Collembola of the genus *Protaphorura* Absolon, 1901 (Onychiuridae) in the Eastern Palearctic: morphology, distribution, identification key

**DOI:** 10.3897/zookeys.620.9372

**Published:** 2016-09-29

**Authors:** Igor Kaprus’, Wanda Weiner, Grzegorz Paśnik

**Affiliations:** 1 State Museum of Natural History, Ukrainian National Academy of Sciences, Teatral’na St. 18, UA-79008 L’viv, Ukraine; 2 Institute of Systematics and Evolution of Animals, Polish Academy of Sciences, Sławkowska 17, 31-016 Kraków, Poland

**Keywords:** Protaphorurini, taxonomy, chaetotaxy, new species, redescription, Siberia, Far East

## Abstract

Seven new species, *Protaphorura jernika* **sp. n.**, *Protaphorura abscondita* **sp. n.**, *Protaphorura tuvinica* **sp. n.**, *Protaphorura vasilinae* **sp. n.**, *Protaphorura sayanica* **sp. n.**, *Protaphorura oligopseudocellata* **sp. n.** and *Protaphorura nikolai* **sp. n.** from different habitats of the southern Siberia and Far East of Russia, are described. *Protaphorura ombrophila* (Stach, 1960) is redescribed based on the type specimens. These species differ one from other and from all known species by dorsal and ventral pseudocellar formulae, number of pseudocelli on subcoxae 1 of legs I–III, parapseudocellar formula, chaetotaxy of body, structure of claw, size of postantennal organ and body length. Geographical distribution of all known *Protaphorura* species of Eastern Palearctic was analysed and an identification key to 50 species was provided.

## Introduction

The genus *Protaphorura* Absolon, 1901, widespread throughout Holarctic, is the most diverse taxon with almost 140 species described to date (Bellinger et al. 2016, Parimuchová and Kováč 2016), forty three of which is known from the Eastern Palearctic (Martynova 1976, Pomorski and Kaprus’ 2007, Kaprus’ and Pomorski 2008, Kaprus’ et al. 2014, Gulgenova and Potapov 2013, Sun, Wu and Gao 2013, Sun, Zhang and Wu 2013, Babenko and Kaprus’ 2014, Sun, Chang and Wu 2015 etc.). The boundaries of the Eastern Palearctic region we determined conventionally from the Ural Mountains and Caspian Sea to Japan Islands and Bering Strait. Siberia, which occupies most of the Eastern Palearctic, continues to be one of the poorly studied geographical regions. The results of this study allow to discover seven new species of *Protaphorura*. Additionally, *Protaphorura ombrophila* (Stach, 1960) is redescribed from Afghanistan, using the type material deposited in the Institute of Systematics and Evolution of Animals, Polish Academy of Sciences in Kraków (Poland). The present paper aims to provide a critical evaluation of all known *Protaphorura* species of the Eastern Palearctic.

## Material and methods

Material of *Protaphorura* species was collected by the soil samples method. Samples were extracted using Berlese–Tullgren funnels. Specimens of new species were collected by Dr. Sophya Stebaeva (Severtsov Institute of Ecology and Evolution, Russian Academy of Sciences, Moscow) in southern Siberia from 1972 to 1994, Dr. Elena Sleptsova (North eastern Federal University in Yakutsk, Russia) in the north eastern Altai in 2002 and Dr. Nikolay Ryabinin (Institute of Water and Ecological Problems, Far Eastern Branch of Russian Academy of Sciences, Khabarovsk) in the Far East of Russia in 2011. Specimens were mounted in Faure’s medium, after clearing in lactophenol, and were studied using Olympus and Leica microscopes. Material is housed in the State Museum of Natural History, Ukrainian National Academy of Sciences, L’viv, Ukraine (SNHM), Institute of Systematics and Evolution of Animals, Polish Academy of Sciences, Kraków, Poland (ISEA) and Moscow Pedagogical State University, Russia (MPSU).

The studied type materials of *Protaphorura ombrophila* (Stach, 1960) are deposited in the Institute of Systematics and Evolution of Animals, Polish Academy of Sciences (Kraków).

Morphological terms. Labial types are named after Fjellberg (1999). Labium areas and chaetal nomenclature follow Massoud (1967) and D’Haese (2003). Tibiotarsal formula is presented after Deharveng (1983). Chaetae on furcal area are notated after Weiner (1996). Chaetae on anal valves are named following Yoshii (1996). Chaetae formula on thoracic tergum I is notated after Gisin (1952).

**Abbreviations used in descriptions**:

Abd. abdominal segments


Ant. antennal segments


AIIIO sensory organ of Ant. III


AS anal spines


pso pseudocellus


ms s-microchaeta


MVO male ventral organ


PAO postantennal organ


psp pseudopore


psx parapseudocellus


Th. thoracic segments


VT ventral tube


1^m^ single psx or psp in medial position.

## Species descriptions

### Protaphorura abscondita sp. n.

Taxon classificationAnimaliaCollembolaOnychiuridae

http://zoobank.org/BC9EAE06-D98C-4A03-964D-2C6035DA71B6

Figs 1–9 , 58

#### Type material.

Holotype (female): Russia, Siberia, Krasnoyarsk Territory, Achinsk Province, 7 km from Nazarovo, steppe meadow, soil, ca 400 m alt., 57°02'N, 90°39'E, 14.VII.1987, leg. S.K. Stebaeva (SNHM). Paratypes: 8 males and 10 females, same data as holotype (SNHM – 7 paratypes: 1 male and 6 females, ISEA – 6 paratypes: 5 males and 1 female, MSPU – 5 paratypes: 2 males and 3 females).

#### Diagnosis.

PAO with 20–23 simple vesicles. Pso formula dorsally 32/033/33343, ventrally 1/000/0000, subcoxae 1 of I–III legs with 1,1,1 pso respectively. Submedial pso a and b on Abd. terga I–II located close together. Psx formula on Abd. sterna: 111100. Th. tergum I with 12–15+12–15 chaetae, chaeta m present. Chaetae s' present on Abd. terga I–III. Manubrial field with 12 chaetae in 3 rows. Claw without lateral denticles.

#### Description.

**Figures 1–9.**
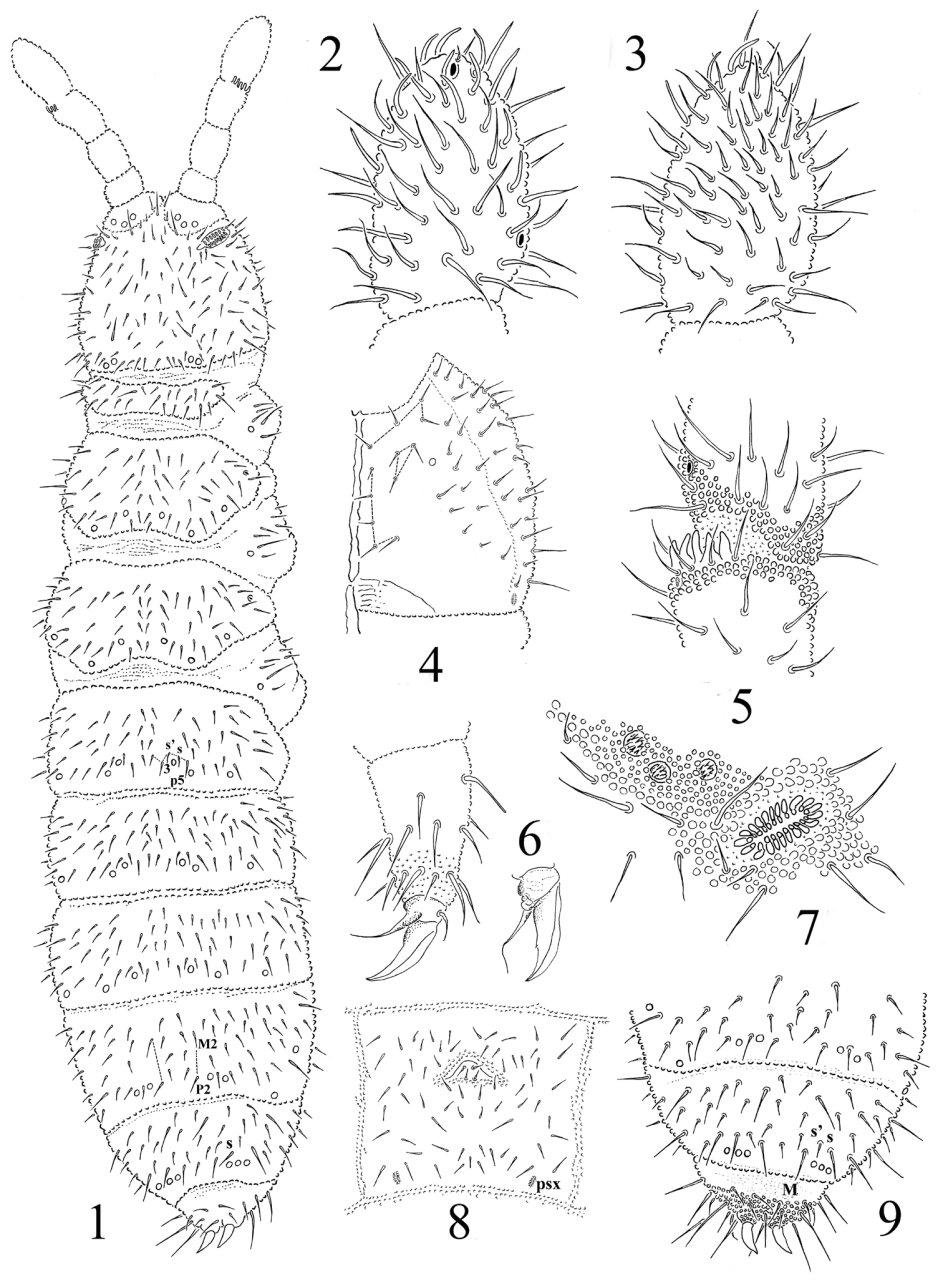
*Protaphorura abscondita*: **1** habitus and dorsal chaetotaxy **2** dorsal side of Ant. IV **3** ventral side of Ant. IV **4** right part of head ventrally **5** AIIIO **6** tibiotarsal chaetotaxy and claw of leg III **7** PAO and anterior cephalic pseudocelli **8** chaetotaxy of Abd. sternum IV **9** chaetotaxy of Abd. terga IVVI.

Holotype (female) length 1.2 mm, length of paratypes: 0.9–1.1 mm (males) and 1.0–1.3 mm (females). Shape of body typical of the genus: cylindrical with strong AS on distinct papillae (Fig. 1). Colour in alcohol yellowish-white. Granulation more or less uniform, distinct. Usually 10–11 grains around each pso.

Antennae approximately as long as head, their base well marked. Ant. I with 10–11 chaetae, Ant. II with 17–18 chaetae. AIIIO consisting of 5 guard chaetae, 5 papillae, 2 smooth sensory rods, 2 straight and granulated sensory clubs, ventro-lateral microsensillum present (Fig. 5). Ant. IV with subapical organite in unprotected cavity without clear cuticular papilla. Microsensillum on Ant. IV in usual position above second proximal row of chaetae (Fig. 2). Ventrally Ant. IV with numerous chaetae (ca. 58–65) (Fig. 3). Ant. IV with 9–11 well-differentiated sensilla (Fig. 2, 3).

PAO of small length with 20–23 simple vesicles (Fig. 7). Labral formula of chaetae: 4/342. Maxillary outer lobe with simple palp, basal chaeta and with two sublobal hairs. Labial palp of type A. Labium with 6 proximal, 4 basomedian (E, F, G, and f), and 6 basolateral chaetae (a, b, c, d, e, e’). Papillae A-E with 1, 4, 0, 3, 3 guard chaetae respectively.

Pso formula dorsally 32/033/33343, ventrally 1/000/0000. Subcoxae 1 of I–III legs with one pso and one psx each. Submedial pso a and b on Abd. terga I–II located close together, i.e. closer than on Abd. tergum III, both set posteriorly to macrochaeta p5. Psx present on Abd. sterna I–IV (psx formula 0/000/111100). Psp formula dorsally 0/011/1111, ventrally 0/111/01^m^1^m^1^m^, coxae with 1 psp each.

Dorsal chaetotaxy rather symmetrical, as in Fig. 1, 4 and 9. Dorsal chaetae poorly differentiated into macrochaetae and microchaetae. Sensory chaetae s distinct on body. On head p2 chaetae on the same level as p1 and p3. Chaetae p6 on head located anterior to pso b. Th. tergum I with 12–15+12–15 chaetae, chaeta m present (chaetotaxy type i2–3m). Both Th. terga II and III with lateral microsensilla and with 5+5 or 6+6 axial microchaetae. Chaetae s' present on Abd. terga I–III, on Abd. tergum V present or absent. On Abd. tergum IV in axial area between M2 and P2 macrochaetae located 7– 8 chaetae, medial chaeta m0 present (rarely absent) (Fig. 1). Abd. tergum V usually with 1–2 unpaired microchaeta m0 and p0 (sometimes m0 absent) (Fig. 1). Abd. tergum VI with 1–2 medial chaetae a0 and m0 (often a0 absent). Relative position of prespinal microchaetae usually of subparallel type (Fig. 9). M/s ratio on Abd. tergum V as 10.5–11.4/9.0–9.5, (AS = 10). AS 1.2–1.3 times longer than inner edge of claw and 2.9–3.0 times longer than their basal diameter.

Chaetotaxy of ventral side of head as in Fig. 4. Perilabial area with 4+4 a-chaetae (Fig. 4). Postlabial chaetae 5+5 along ventral groove. Th. sterna I–III with 1+1, 2+2, 2+2 chaetae respectively. VT with ca. 7–9+7–9 chaetae, and 2 chaetae at base. Chaetotaxy of Abd. sternum IV as in Fig. 8. Furcal rudiment: cuticular fold (located near the middle of sternum) with 2+2 dental microchaetae in 2 rows. Chaetotaxy of manubrial field rather stable: 4 chaetae present in ma-row, 4 chaetae in mm-row and 4 chaetae in mp-row (Fig. 8). MVO absent. Each lateral anal valves with a0, 2a1 and 1-2a2; upper anal valve with chaetae a0, 2a2, 2b1, 2b2, c0, 2c1 and 2c2 (as in *Protaphorura jernika*, Fig. 58).

Subcoxae 1 of I, II and III legs with 5, 7, 6 chaetae, subcoxae 2 with 1, 5, 5, coxae with 3, 10, 14, trochanters with 11, 11, 10, femora with 17 each, tibiotarsi with four rows of chaetae (distal whorl (A+T)+B+C): 11+8+3, 11+8+3, 11+8+4 chaetae respectively. Claw with very small (rarely without) denticle in 1/2 of inner edge of claw (Fig. 6). Empodial appendage of same length as inner edge of claw, without basal lamella (Fig. 6).

#### Etymology.

The name of the new species refers to the Latin *absconditus* (hidden, concealed).

#### Discussion.

*Protaphorura abscondita* sp. n. is characterized by a unique formula of dorsal pso: 2+2 posterior cephalic pso, 3+3 pso on Th. terga II and III and Abd. tergum V. Among seven known species with 3+3 pso on Th. terga II and III, the new species is most similar to the siberian *Protaphorura tundricola* (Martynova, 1976), *Protaphorura submersa* Kaprus’ & Pomorski, 2008 and *Protaphorura merita* Kaprus’ & Pomorski, 2008 due to number of pso on Abd. terga. *Protaphorura abscondita* sp. n. differs from all these species by the 9-11 well differentiated sensilla on Ant. IV. Additionally, it differs from *Protaphorura merita* by the absence of cauliflower like papilla on the tip of antenna and 1+1 ventral pso in posterolateral position on head. From *Protaphorura submersa*, the new species differs by having 3 pso on the base of antennae (4(5) pso in *Protaphorura submersa*) and from *Protaphorura tundricola* by relative position of prespinal microchaetae on Abd.6 (distinctly convergent type in *Protaphorura tundricola* and subparallel type in *Protaphorura abscondita*).

### Protaphorura jernika sp. n.

Taxon classificationAnimaliaCollembolaOnychiuridae

http://zoobank.org/A4590F99-71B6-4923-8178-696819C5AD5F

Figs 10–17 , 58

#### Type material.

Holotype (female): Russia, N-E Altai, Turochak Region, Altyn-Tu Mt. Ridge, Archa Mt, mountain shrub tundra (=jernik tundra) with *Betula rotundifolia*, moss, 1700–1800 m alt., 51°31'N, 87°27'E, 9.VIII.2002, leg. E.V. Sleptsova (ISEA). Paratypes: 2 males, same data as holotype (SNHM).

#### Diagnosis.

PAO with 39–44 simple vesicles. Pso formula dorsally 32/033/33342, ventrally 2/000/0001, subcoxae 1 of I–III legs with 1,1,1 pso respectively. Submedial pso a and b on Abd. terga I–II located far from each other. Psx formula on Abd. sterna: 111000. Th. tergum I with 12–15+12–15 chaetae, chaeta m present. Chaetae s' absent on Abd. terga I–III and V. Manubrial field with 16–17 chaetae in 4 rows. Claw without lateral denticles.

#### Description.

**Figures 10–17.**
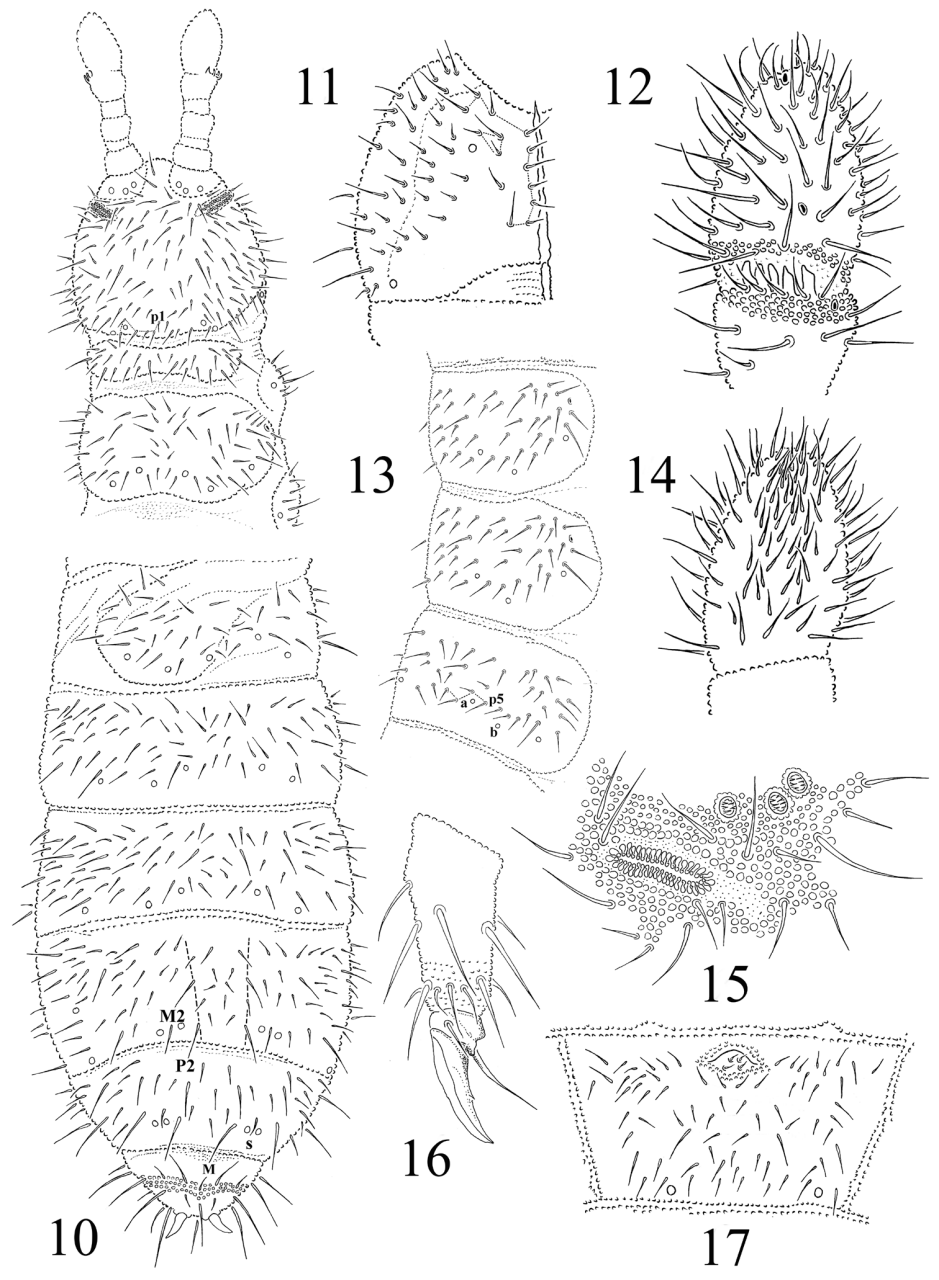
*Protaphorura jernika*: **10** habitus and dorsal chaetotaxy **11** left part of head ventrally **12** dorsal side of Ant. III–IV **13** chaetotaxy of Th. terga III aand Abd. tergum I **14** ventral side of Ant. IV **15** PAO and anterior cephalic pseudocelli **16** tibiotarsal chaetotaxy and claw of leg III **17** chaetotaxy of Abd. sternum IV.

Holotype (female) length 1.8 mm, length of paratypes: 1.4 mm (males). Shape of body typical for the genus: cylindrical with strong AS on distinct papillae (Fig. 10). Colour in alcohol yellowish-white. Granulation distinct, usually slightly coarser on head, Abd. tergum VI and around pso. Usually 9–11 grains around each pso.

Antennae slightly shorter than head, their base well marked. Ant. I with 10 chaetae, Ant. II with 18 chaetae. AIIIO consisting of 5 guard chaetae, 5 papillae, 2 smooth sensory rods, 2 straight and granulated sensory clubs, ventro-lateral microsensillum present (Fig. 12). Ant. IV with subapical organite in unprotected cavity without clear cuticular papilla. Microsensillum on Ant. IV in usual position on the level of second proximal row of chaetae. Ant. IV ventrally with very numerous chaetae (ca. 70–75) (Fig. 14). Sensilla indistinct on Ant. IV.

PAO of middle length with 39–44 simple vesicles (Fig. 15). Labral formula of chaetae: 4/342. Maxillary outer lobe with simple palp, basal chaeta and with two sublobal hairs. Labial palp of type A. Labium with 6 proximal, 4 basomedian (E, F, G, and f), and 6 basolateral chaetae (a, b, c, d, e, e’). Papillae A-E with 1, 4, 0, 3, 3 guard chaetae respectively.

Pso formula dorsally 32/033/33342, ventrally 2/000/0001 (Figs 10, 11, 13, 17). Subcoxae 1 of I–III legs with one pso and one psx each. Submedial pso a and b on Abd. terga I–II located far apart, i.e. on similar distance as on Abd. tergum III (Fig. 13). Psx present on Abd. sterna I–III (psx formula 0/000/111000). Psp formula dorsally 0/011/1111, ventrally 0/111/01^m^1^m^1^m^, coxae with 1 psp each.

Dorsal chaetotaxy, slightly asymmetrical and rather plurichaetotic, as in Figs 10 and 13. Dorsal chaetae rather well differentiated into macrochaetae and microchaetae. Sensory chaetae s indistinct on body. On head p1 chaetae are displaced forward in relation to p2–p4 (Fig. 10). Chaetae p6 on head located between pso a and b. Th. tergum I with 12–15+12–15 chaetae, chaeta m present (chaetotaxy type i2–3m). Both Th. terga II and III with lateral microsensilla and with 5+5 or 6+6 axial microchaetae. Chaetae s' absent on Abd. terga I–III and V. On Abd. tergum IV in axial area between M2 and P2 macrochaetae located 8–12 chaetae, medial chaeta m0 present (rarely absent) (Fig. 10). Abd. tergum V usually with 1–2 unpaired microchaeta m0 and p0 (sometimes m0 absent) (Fig. 10). Abd. tergum VI with 1–2 medial chaetae a0 and m0 (rarely a0 absent). Relative position of prespinal microchaetae usually of parallel type (Fig. 10). M/s ratio on Abd. tergum V as 13.6–17.6/5.6–6.9 (AS = 10). AS 1.1 times longer then inner edge of claw and 2.6 times longer then their basal diameter.

Chaetotaxy of ventral side of head as in Fig. 11. Perilabial area with 4+4 a-chaetae (Fig. 11). Postlabial chaetae 5-6+5-6 along ventral groove. Th. sterna I–III with 0+0, 1+1, 1+1 chaetae respectively. VT with ca. 8–9+8–10 chaetae and 1+2 chaetae at base. Furcal rudiment: cuticular fold (located on the anterior edge of sternum) with 2+2 dental microchaetae in 2 rows. Chaetotaxy of manubrial field: 4 chaetae present in ma-row, 4 chaetae in mm’-row, 4 chaetae in mm-row and 4–5 chaetae in mp-row (Fig. 17). MVO absent. Each lateral anal valves with a0, 2a1 and 2a2; upper anal valve with chaetae a0, 2a2, 2b1, 2b2, c0, 2c1 and 2c2 (Fig. 58).

Subcoxae 1 of I, II and III legs with 5–7, 6–8, 5–6 chaetae, subcoxae 2 with 1, 5, 5, coxae with 3, 8, 14, trochanters with 11, 11, 10, femora with 19 each, tibiotarsi with four rows of chaetae (distal whorl (A+T)+B+C): 11+8+3, 11+8+3, 11+8+4 chaetae respectively. Claw with strong denticle in 1/2 of inner edge of claw (Fig. 16). Empodial appendage of same length as inner edge of claw, without basal lamella (Fig. 16).

#### Etymology.

The name of the new species refers to the Russian “jernik” (= shrub tundra or tundra with dwarf birch).

#### Discussion.

*Protaphorura jernika* sp. n. belongs to the group of *Protaphorura* species with pseudocelli on subcoxa 1 of all legs and 2+2 pso ventrally on head. By the presence of 1+1 pso on Abd. sternum IV, the new species is similar to the *Protaphorura vasilinae* sp. n. Both species differ only in the formula of dorsal pso and ventral psx on Abd. sterna: the former has 32/033/33342 pso and 111000 psx whereas the latter 32/022/33332 pso and 110001^m^ psx (see also diagnosis of *Protaphorura vasilinae* sp. n.). *Protaphorura jernika* sp. n. differs from other two Eastern Palearctic representatives of this group, *Protaphorura merita* Kaprus’ & Pomorski, 2008 and *Protaphorura buryatica* Gulgenova & Potapov, 2013 by dorsal pso formula (32/033/33342 in the new species vs 32(3)/012/33342 in *buryatica* and 43/02(3)2(3)/3335(4,6)3(4) in *merita*), by the presence of 1+1 pso on abd. sternum IV in the new species and lack in the both other, by the number of vesicles in PAO (39-44 in the new species, 12-13 in *buryatica* and 16-22 in *buryatica*).

### Protaphorura nikolai sp. n.

Taxon classificationAnimaliaCollembolaOnychiuridae

http://zoobank.org/AA913DC8-EE15-44C5-AD7E-5092B2F8F207

Figs 18–25 , 58

#### Type material.

Holotype (male): Russia, Primorsky Krai, Khasansky district, Barabash village, mixed forest with *Quercus*, *Acer* and *Juglans*, in soil and leave litter, 9.VII.2011, leg. N.A. Ryabinin (SNHM). Paratypes: 6 males and 6 females, same data as holotype (SNHM – 9 paratypes: 5 male and 4 females, ISEA – 3 paratypes: 1 male and 2 females).

#### Diagnosis.

PAO with 29–36 simple vesicles. Pso formula dorsally 33/022/33342, ventrally 1/000/0000, subcoxae 1 of I–III legs with 1,0,0 pso respectively. Submedial pso a and b on Abd. terga I–II located close together. Psx formula on Abd. sterna: 100000. Th. tergum I with 11–12+11–12 chaetae, chaeta m present. Chaetae s' absent on Abd. terga I–III and V. Manubrial field with 14–15 chaetae in 3 rows. Claw without lateral denticles.

#### Description.

**Figures 18–25.**
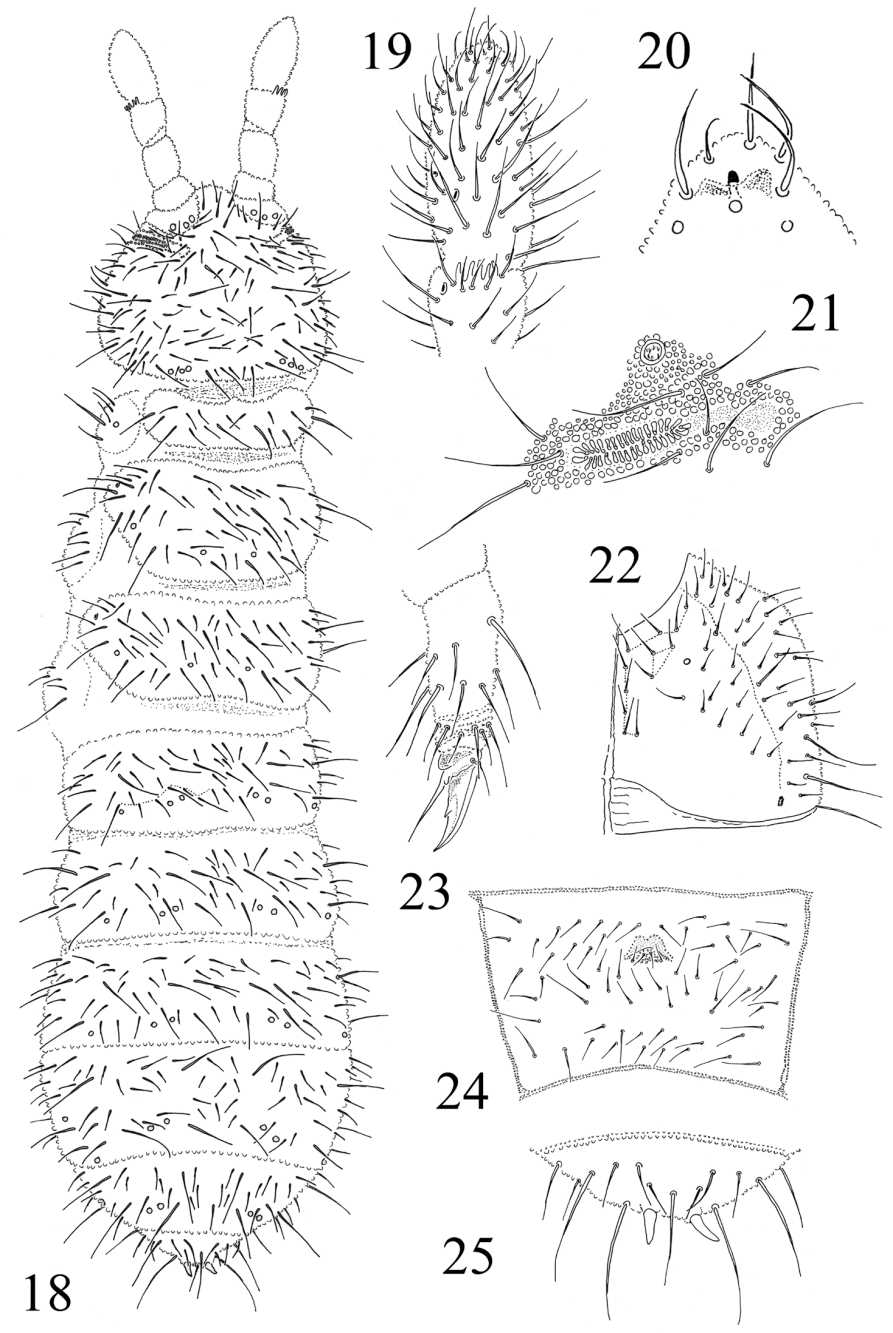
*Protaphorura nikolai*: **18** habitus and dorsal chaetotaxy **19** dorsal side of Ant. III–IV **20** tip of Ant. IV **21** PAO and anterior cephalic pseudocelli **22** right part of head ventrally **23** tibiotarsal chaetotaxy and claw of leg III **24** chaetotaxy of Abd. sternum IV **25** chaetotaxy of Abd. tergum VI.

Holotype (male) length 1.5 mm, length of paratypes: 1.45–1.55 mm (males) and 1.58–1.72 mm (females). Shape of body typical for the genus: cylindrical with strong AS on distinct papillae (Fig. 18). Colour in alcohol yellowish-white. Granulation more or less uniform, distinct. Usually 12–14 grains around each pso.

Antennae approximately as long as head, their base well marked. Ant. I with 11–12 chaetae, Ant. II with 17–18 chaetae. AIIIO consisting of 5 guard chaetae, 5 papillae, 2 smooth sensory rods, 2 straight and granulated sensory clubs, ventro-lateral microsensillum present (Fig. 19). Ant. IV with subapical organite in cavity protected by cuticular papillae (Fig. 20). Microsensillum on Ant. IV situated on level or below of second proximal row of chaetae. Ventrally Ant. IV with numerous chaetae (ca. 68–72). Ant. IV without differentiated sensilla (Fig. 19).

PAO is relatively small with 29–36 simple vesicles (Fig. 21). Labral formula of chaetae: 4/342. Maxillary outer lobe with simple palp, basal chaeta and with two sublobal hairs. Labial palp of type A. Labium with 7 proximal, 4 basomedian (E, F, G, and f), and 6 basolateral chaetae (a, b, c, d, e, e’). Papillae A-E with 1, 4, 0, 3, 3 guard chaetae respectively.

Pso formula dorsally 33/022/33342, ventrally 1/000/0000 (Figs 18, 22, 24). Subcoxae1 of legs I, II and III with 1,0,0 pso respectively. Psx on subcoxae1 of legs I, II and III absent. Submedial pso a and b on Abd. terga I–II located close together, i.e. much closer than on Abd. tergum III, both set posteriorly to macrochaeta p5 (Fig. 18). Ventral psx formula 1/000/100000). Psp formula dorsally 0/011/1111, ventrally 0/111/01^m^1^m^1^m^, coxae with 1 psp each.

Dorsal chaetotaxy slightly asymmerical, chaetae well differentiated into macrochaetae, mesochaetae and microchaetae as in Fig. 18. Sensory chaetae s indistinct on body. On head p2 chaetae on same level as p1 and p3. Chaetae p6 on head located anterior to pso b (Fig. 18). Th. tergum I with 11–12+11–12 chaetae, chaeta m present (chaetotaxy type i2–3m). Both Th. terga II and III with lateral microsensilla and with 4+4 or 5+5 axial microchaetae. Chaetae s' absent on Abd. terga I–III and V. On Abd. tergum IV in axial area between M2 and P2 macrochaetae located 9–11 chaetae, medial chaeta m0 present or absent, p0 present or absent (Fig. 18). Abd. tergum V usually with 1 unpaired microchaeta p0 (m0 absent) (Fig. 18). Abd. tergum VI with 1 medial chaetae m0. Relative position of prespinal microchaetae of distinctly divergent type (Fig. 25). M/s ratio on Abd. tergum V as 33–40/20–22, (AS = 10). AS 0.6–0.7 times as long as inner edge of claw and 2.0 times longer than their basal diameter.

Chaetotaxy of ventral side of head as in Fig. 22. Perilabial area with 4+4 a-chaetae. Postlabial chaetae 5+5 along ventral groove. Th. sterna I–III with 1+1, 2+2, 2+2 chaetae respectively. VT with ca. 8–9+8–9 chaetae, and 2(1)+2(1) chaetae at base. Chaetotaxy of Abd. sternum IV as in Fig. 22. Furcal rudiment: cuticular fold (located near the middle of sternum) with 2+2 dental microchaetae in 2 rows. Chaetotaxy of manubrial field: 4 chaetae present in ma-row, 6-7 chaetae in mm-row and 4 chaetae in mp-row (Fig. 24). MVO absent. Each lateral anal valves with a0, 2a1 and 2a2; upper anal valve with chaetae a0, 2a2, 2b1, 2b2, c0, 2c1 and 2c2 (as in *Protaphorura jernika*, Fig. 58).

Subcoxae 1 of I, II and III legs with 5–6, 6–7 and 5–6 chaetae respectively, subcoxae 2 with 1, 5, 5, coxae with 3, 11, 13, trochanters with 11, 11, 10, femora with 21, 21, 18, tibiotarsi with four rows of chaetae (distal whorl (A+T)+B+C): 11+8+4, 11+8+4, 11+8+4–5 chaetae respectively. Claw with very strong denticle in the 1/2 of inner edge of claw (Fig. 23). Empodial appendage 0,9–1,0 times as long as inner edge of claw, without basal lamella (Fig. 23).

#### Etymology.

The species is cordially dedicated to Russian oribatologist Dr. Nikolay Ryabinin, who collected the type material of new species in Primorsky Krai of Russia.

#### Discussion.

*Protaphorura nikolai* sp. n. belongs to the group of *Protaphorura* species with 1,0,0 pseudocelli on subcoxa 1 of I, II and III legs and 1+1 pso on head ventrally. Among Asiatic species this group includes *Protaphorura zori* (Martynova, 1975 in Martynova & Chelnokov, 1975)(although Martynova did not mention subcoxal pso, the examined by us type has 1,0,0 pso on subcoxae), *Protaphorura brevispinata* (Yosii, 1966), *Protaphorura changbaiensis* Sun, Zhang & Wu, 2013, *Protaphorura mongolica* (Martynova, 1975 in Martynova & Chelnokov, 1975), *Protaphorura sakatoi* (Yosii, 1966) and *Protaphorura maoerensis* Sun, Wu & Gao, 2013. Within this group, it shares dorsal pso formula with *Protaphorura zori* but differs from the latter by the presence of inner denticle on claw, the absence of chaeta a0 on Abd. tergum VI (in *Protaphorura zori* inner denticle absent and chaeta a0 present) and by arrangement of prespinal chaetae (placed divergently in *Protaphorura nikolai* and convergently in *Protaphorura zori*). Perhaps there are other differences in the morphology of these two species, but *Protaphorura zori* is not well described and needs more detailed study.

### Protaphorura oligopseudocellata sp. n.

Taxon classificationAnimaliaCollembolaOnychiuridae

http://zoobank.org/3FC95D7C-4065-4C63-989F-FBD2B1635E9F

Figs 26–32 , 58

#### Type material.

Holotype (female): Russia, Siberia, Western Sayan, Oiskii Mt. Range, vicinity of weather station Olenya Rechka, mountain tundra with *Betula rotundifolia*, *Salix* sp, *Sphagnum* sp., 1800 m alt., in moss and soil, 52°48'N, 93°13'E, 27.VI.1990, leg. S.K. Stebaeva (SNHM). Paratypes: 3 females and juvenile, same data as holotype (ISEA – 1 paratype, MSPU – 1 paratype and juvenile).

#### Diagnosis.

PAO with 32–34 simple vesicles. Pso formula dorsally 32/011/22232, ventrally 1/000/0000, subcoxae 1 of I–III legs without pso. Psx formula on Abd. sterna: 111000. Th. tergum I with 23–25+23–25 chaetae, one, two or three chaetae m present. Chaetae s' present on Abd. terga I–III and absent or present on Abd. tergum V. Manubrial field with 12–13 chaetae in three rows. Claw with pair of lateral denticles.

#### Description.

**Figures 26–32.**
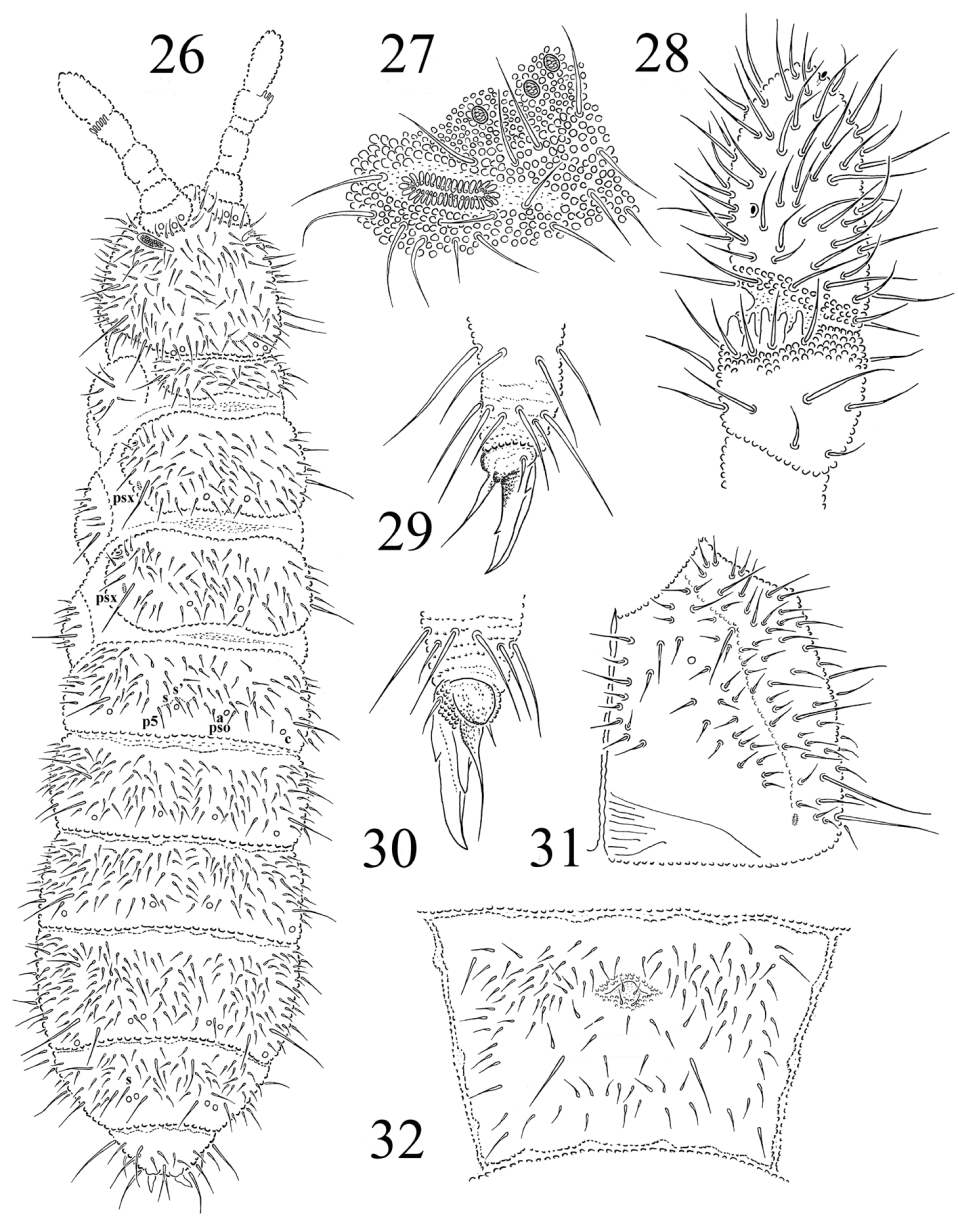
*Protaphorura oligopseudocellata*: **26** habitus and dorsal chaetotaxy **27** PAO and anterior cephalic pseudocelli **28** dorsal side of Ant. IIIIV **29** and **30** distal part of leg III **31** right part of head ventrally **32** chaetotaxy of Abd. sternum IV.

Holotype (female) length 2.2 mm, length of paratypes: 2.0–2.3 mm (females). Shape of body typical of the genus: cylindrical with strong AS on distinct papillae (Fig. 26). Colour in alcohol yellowish-white. Granulation more or less uniform, distinct. Usually 7–10 grains around each pso.

Antennae as long as the head, their base well marked. Ant. I with 11–12 chaetae, Ant. II with 18 chaetae. AIIIO consisting of 5 guard chaetae, 5 papillae, 2 smooth sensory rods, 2 straight and granulated sensory clubs, ventro-lateral microsensillum present (Fig. 28). Ant. IV with subapical organite in unprotected cavity without clear cuticular papilla. Microsensillum on Ant. IV in usual position above second proximal row of chaetae. Ventrally Ant. IV with numerous chaetae (ca. 74–78). Sensilla indistinct on antennal segment IV (Fig. 28).

PAO relatively small, consisting of 32-34 simple vesicles (Fig. 27). Labral formula of chaetae: 4/342. Maxillary outer lobe with simple palp, basal chaeta and with two sublobal hairs. Labial palp of type A. Labium with 7 proximal, 4 basomedian (E, F, G, and f), and 6 basolateral chaetae (a, b, c, d, e, e’). Papillae A-E with 1, 4, 0, 3, 3 guard chaetae respectively.

Pso formula dorsally 32/011/22232, ventrally 1/000/0000 (Figs 26, 31, 32). Subcoxae 1 of I–III legs without pso and with one psx each. Psx formula 1/000/111000. Th. terga II and III with 1+1, 1+1 psx in lateral position (Fig. 26). Psp formula dorsally 0/011/1111, ventrally 0/111/01^m^1^m^1^m^, coxae with 1 psp.

Dorsal chaetotaxy plurichaetotic, usually with some asymmetry, all dorsal chaetae rather short (except macrochaetae), well differentiated into macro- meso- and microchaetae, as in Fig. 26. Sensory chaetae s indistinct on body. On head p2 chaetae are displaced forward in relation to p1 and p3. Chaetae p6 on head located anterior to pseudocelli b (Fig. 26). Th. tergum I with 23–25+23–25 chaetae, 1–3 chaetae m and 1–2 chaetae i present (chaetotaxy type i(1–2)3–4m(1–3)). Both Th. terga II and III with lateral microsensilla and with 6+6 or 7+7 axial microchaetae. Chaetae s' present on Abd. terga I–III and absent or present on Abd. tergum V (Fig. 26). On Abd. tergum IV in axial area between M2 and P2 macrochaetae located 23–24 chaetae, medial chaetae p0 and m0 present (sometimes these chaetae absent). Abd. tergum V with one unpaired microchaeta p0 (Fig. 26). Abd. tergum VI with medial chaetae m0. Relative position of prespinal microchaetae of convergent type (Fig. 26). M/s ratio on Abd. tergum V as 23.5–23.9/15 (AS = 10). AS 0.7–0.8 times as long as inner edge of claw and 2.3 times longer than their basal diameter.

Chaetotaxy of ventral side of head as in Fig. 31. Perilabial area with 4–5+4–5 a-chaetae. Postlabial chaetae 4-5+4-5 along ventral groove. Thoracic sterna I–III with 1+1, 2–3+2–3, 2–3+2–3 chaetae respectively. VT with ca. 10+10 chaetae, and 2–3 chaetae at base. Chaetotaxy of Abd. sternum IV as in Fig. 32. Furcal rudiment: cuticular fold (located near the middle of sternum) with 2+2 dental microchaetae in 2 rows. Chaetotaxy of manubrial field: 4–5 chaetae present in ma-row, 4 chaetae in mm-row, 4 chaetae in mp-row (in adult specimens) (Fig. 32). Each lateral anal valves with a0, 2a1 and 2a2; upper anal valve with chaetae a0, 2a2, 2b1, 2b2, c0, 2c1 and 2c2 (as in *Protaphorura jernika*, Fig. 58).

Subcoxae 1 of I, II and III legs with 6–8, 7–8, 7–9 chaetae, subcoxae 2 with 1, 5, 5, coxae with 4, 10, 15, trochanters with 13, 15, 15, femora with 21, 23, 22–23, tibiotarsi with four rows of chaetae (distal whorl (A+T)+B+C): 11+8+3–4, 11+8+5–6, 11+8+5 chaetae respectively. Claw with strong denticle in 1/2 of inner edge of claw and pair of lateral denticles (Figs 29, 30). Empodial appendage 0.9 times as long as inner edge of claw, without basal lamella (Fig. 29).

#### Etymology.

The name of the new species refers to the Latin *oligo* (a few) and *pseudocellus* (false ocellus) ‒ characteristic structure in Onychiuroidea.

#### Discussion.

*Protaphorura oligopseudocellata* sp. n. is characterized by the reduced number of pso on body dorsally – 32/011/22232. Only four species with 1+1 pso on Th. tergum III is currently known: *Protaphorura januarii* (Weiner, 1977), *Protaphorura stiriaca* (Stach, 1946), *Protaphorura pseudostyriaca* (Loksa, 1964) and *Protaphorura pseudarmata* (Folsom, 1917). The first three species are described from Europe and the last one from North America. Among these species *Protaphorura oligopseudocellata* sp. n. is probably the most similar to *Protaphorura januarii* and *Protaphorura stiriaca* due to the absence of pso on subcoxa 1 of all legs and some similarity of dorsal pso formulae. The new species can be easily distinguished from these species by the number of pso on Abd. terga I–V (22232 in *Protaphorura oligopseudocellata* sp. n., 23232 in *Protaphorura januarii* and 33232 in *Protaphorura stiriaca*), the plurichaetotic chaetotaxy and by the presence of strong lateral denticles on claws and 1+1 pso on head ventrally (lateral denticles and pso absent in *Protaphorura januarii* and *Protaphorura stiriaca*).

### Protaphorura ombrophila

Taxon classificationAnimaliaCollembolaOnychiuridae

(Stach, 1960)

Figs 33–36 , 59

Onychiurus ombrophilus Stach, 1960: 509 – 514, pl. LXV

#### Type material.

Lectotype (female) (by present designation): Afghanistan, ”Tchehel Sotoun” Cave near Jalrayz, W Kabul, with the original label: “Tchehel Sotoun-Höhle (nahe Djalrez), 20.III.1959”, leg. Dr. K. Lindberg. Paralectotypes: 1 male and 8 females, same data as lectotype.

#### Redescription.

Lectotype (female) length 1.9 mm, length of paralectotypes: 1.8 mm (male) and 1.8–2.2 mm (females). Shape of body typical of the genus: cylindrical with strong AS on distinct papillae. Colour in alcohol white. Granulation more or less uniform, distinct. Usually 11–13 grains around each pso.

Antennae slightly shorter than head, their base well marked. Ant. I with 10 chaetae, Ant. II with 16–18 chaetae. AIIIO consisting of 5 guard chaetae, 5 papillae, 2 smooth sensory rods, 2 straight and granulated sensory clubs, ventro-lateral microsensillum present. Ant. IV with subapical organite in unprotected cavity without clear cuticular papilla. Microsensillum on Ant. IV in usual position above second proximal row of chaetae. Sensilla indistinct on Ant. IV.

PAO of middle length, consisting of 24–38 simple vesicles. Labral formula of chaetae: 4/342. Maxillary outer lobe with simple palp, basal chaeta and with two sublobal hairs. Labial palp of type A. Chaetotaxy of labium invisible.

**Figures 33–36.**
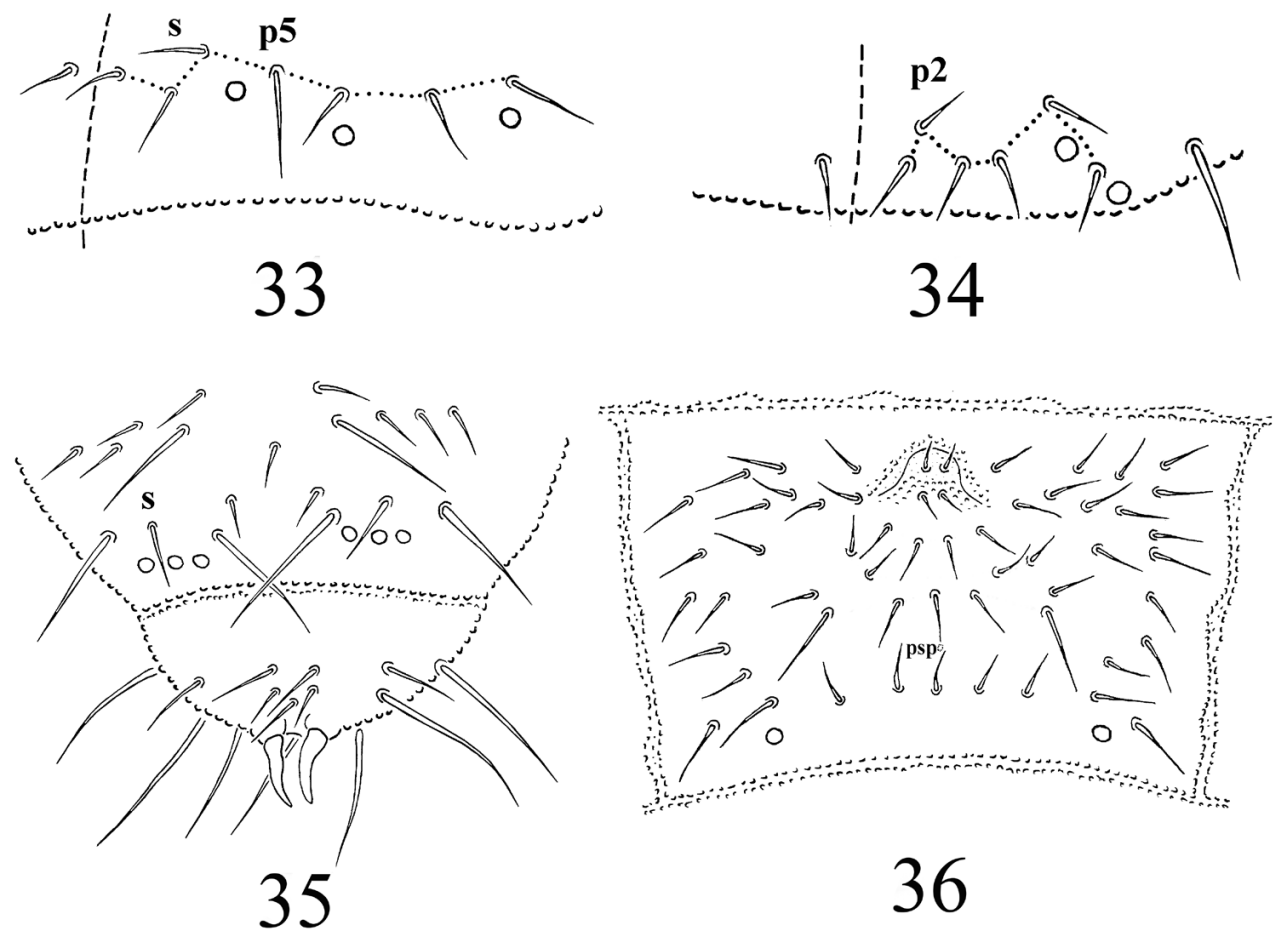
*Protaphorura ombrophila*: **33** position of pso and p-chaetae in midsection of Abd. tergum I **34** position of p-chaetae on posterior margin of head **35** chaetotaxy of Abd. terga V–VI **36** chaetotaxy of Abd. sternum IV.

Pso formula dorsally 32/022(3)/33(2)3(2)43, ventrally 2/000/0001 (Figs 33–36). Subcoxae 1 of I–III legs without pso. Submedial pseudocelli a and b on Abd. terga I–II located far apart, i.e. on similar distance as on Abd. tergum III (Fig. 33). Psx formula 0/000/11?00?.

Dorsal chaetotaxy rather symmetrical. Dorsal chaetae well differentiated into macrochaetae and microchaetae. On head p2 chaetae are displaced forward in relation to p1 and p3 (Fig. 34). Chaetae p6 on head located between pseudocelli a and b. Th. tergum I with 8–10+8–10 chaetae, chaeta m absent (chaetotaxy type i2-). Both Th. terga II and III with lateral microsensilla. Chaetae s' absent on Abd. terga I–III and V (Fig. 35). On Abd. tergum IV in axial area between M2 and P2 macrochaetae located 6–7 chaetae, medial chaeta m0 present. Abd. tergum V usually with 1 unpaired microchaeta m0 (p0 absent) (Fig. 35). Abd. tergum VI with 1 medial chaetae m0. Relative position of prespinal microchaetae usually divergent or parallel type (Fig. 35). M/s ratio on Abd. tergum V as 18.2/8.8 (AS = 10). AS 0.8–0.9 times as long as inner edge of claw and 2.8-3.4 times longer then their basal diameter.

Perilabial area with 4+4 a-chaetae. Th. sterna I–III without chaetae. VT with ca. 8–9+8–9 chaetae, and 1 chaetae at base. Furcal rudiment: cuticular fold (located on the anterior edge of the sternum) with 2+2 dental microchaetae in 2 rows. Chaetotaxy of manubrial field: 4 chaetae present in ma-row, 2 chaetae in mm’ -row, 4 chaetae in mm-row and 5 chaetae in mp-row (Fig. 36). MVO absent. Each lateral anal valves with a0 and 2a1 (a2 absent); upper anal valve with chaetae a0, 2a2, 2b1, 2b2, c0, 2c1 and 2c2 (as in *Protaphorura vasilinae*, Fig. 59).

Subcoxae 1 of I, II and III legs with 5, 6, 5–6 chaetae, tibiotarsi with four rows of chaetae (distal whorl (A+T)+B+C): 11+8+3, 11+8+3, 11+8+4 chaetae respectively. Claw with very small denticle in 1/2 of inner edge of claw. Empodial appendage 0.7–0.8 times as long as inner edge of claw, without basal lamella.

#### Remarks.

*Protaphorura ombrophila* has been described by Stach (1960) from Afghanistan about 55 years ago, when many important diagnostic characters remained unknown. Latter Yosii (1966), during his research on some Collembola of Afghanistan, India and Ceylon, discovered three females of the species in Afghanistan and wrote: “They (i.e. *Protaphorura ombrophila*) coincide fairly well with the detailed description of Stach. However, the posterior margin of head has 3+3, 3+2 and 2+2 pseudocelli. In other respects no difference is to be found”. Parimuchová and Kováč in their recent publication (2016) devoted to the critical analysis of Palearctic species of the genus *Protaphorura* and assigned this species to the group “species dubia”. Here we present first redescription of *Protaphorura ombrophila* based on characters currently used in taxonomy of *Protaphorura*. See also the discussion in *Protaphorura tuvinica* sp. n.

### Protaphorura sayanica sp. n.

Taxon classificationAnimaliaCollembolaOnychiuridae

http://zoobank.org/6F4A0F37-3673-4C0F-BB2D-95B05B6799E2

Figs 37–44 , 58

#### Type material.

Holotype (male): Russia, Siberia, Western Sayan, Oiskii Mt. Range, vicinity of weather station Olenya Rechka, mountain tundra, 1800 m alt., in moss and soil, 52°48'N, 93°13'E, 10.VII.1990, leg. S.K. Stebaeva (SNHM). Paratypes: 2 females and 2 juveniles, same data as holotype (SNHM – 1 paratype female, ISEA – 1 paratype female and 2 juveniles); 2 females: Russia, Krasnoyarsk Territory, Khakasia, Kuznetskii Alatau Mt. Range, ca 5 km NW of settl. Kommunar, mountain tundra with *Dryas oxyodontha*, 1500 m alt., 54°20'N, 89°17'E, 24.VII.1990, leg. S.K. Stebaeva (ISEA); 2 males, female and 2 juveniles: Russia, Kuznetskii Alatau Mt. Range, Kemerovo Prov., 10 km NW of Mezhdurechensk, mixed taiga with rich herbaceous cover, under *Abies sibirica*, soil, 500-600 m alt., 53°45'N, 88°00'E, 1.VII.1982, leg. S.K. Stebaeva (SNHM); male: Russia, Salair Range, 130 km SE of Novosibirsk, 11 km N of Mirnyi, chern forest, 500 m alt., soil, 54°38'N, 84°45'E, 7.VI.1972, leg. S.K. Stebaeva (MPSU); female subadult: Russia, West Siberia, 25 km S of Novosibirsk, Akademgorodok, glade in birch forest, soil, 400 m alt., 54°49'N, 83°08'E, 7.X.1994, leg. S.K. Stebaeva (SNHM).

#### Diagnosis.

PAO with 41–48 simple vesicles. Pso formula dorsally 32/022/33343, ventrally 1/000/0000, subcoxae 1 of I–III legs with 1,1,1 pso respectively. Submedial pso a and b on Abd. terga I–II located far apart. Psx formula on Abd. sterna: 111101^m^. Th. tergum I with 18–21+18–21 chaetae, one or two chaetae m present. Chaetae s' present on Abd. terga I–III and V. Manubrial field with 14 chaetae in three rows. Claw with pair of lateral denticles.

#### Description.

**Figures 37–44.**
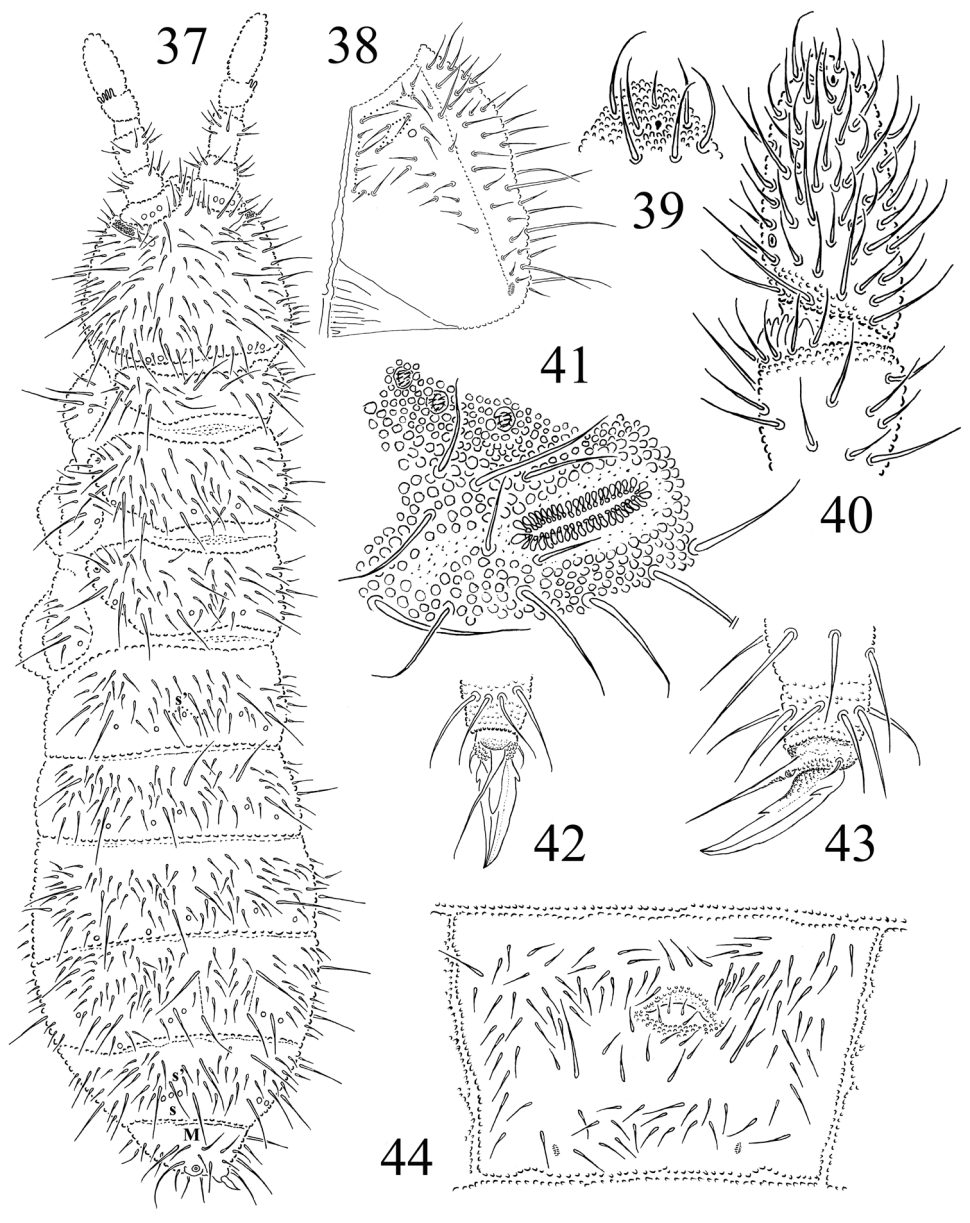
*Protaphorura sayanica*: **37** habitus and dorsal chaetotaxy **38** right part of head ventrally **39** tip of Ant. IV **40** dorsal side of Ant. IIIIV **41** PAO and anterior cephalic pseudocelli **42** and **43** distal part of leg III **44** chaetotaxy of Abd. sternum IV.

Holotype (male) length 2.7 mm, length of paratypes: 2.7–2.9 mm (females). Other specimens length: 2.62.7 mm males and 2.8 mm female. Shape of body typical of the genus: cylindrical with strong AS on distinct papillae (Fig. 37). Colour in alcohol yellowish-white. Granulation more or less uniform, distinct. Usually 7–9 grains around each pso.

Antennae as long as the head, their base well marked. Ant. I with 11 chaetae, Ant. II with 16–19 chaetae. AIIIO consisting of 5 guard chaetae, 5 papillae, 2 smooth sensory rods, 2 straight and granulated sensory clubs, ventro-lateral microsensillum present (Fig. 40). Ant. IV with subapical organite in unprotected cavity without clear cuticular papilla (Fig. 39). Microsensillum on Ant. IV in usual position above second proximal row of chaetae. Ventrally Ant. IV with numerous chaetae (ca. 65–70) (Fig. 40). Sensilla indistinct on Ant. IV (Fig. 40).

PAO large, consisting of 41–48 simple vesicles (Fig. 41). Labral formula of chaetae: 4/342. Maxillary outer lobe with simple palp, basal chaeta and with two sublobal hairs. Labial palp of type A. Labium with 7 proximal, 4 basomedian (E, F, G, and f), and 6 basolateral chaetae (a, b, c, d, e, e’). Papillae A-E with 1, 4, 0, 3, 3 guard chaetae respectively.

Pso formula dorsally 32/022/33343, ventrally 1/000/0000 (Figs 37, 38, 44). Subcoxae 1 of I–III legs with one pso and one psx each. Submedial pso a and b on Abd. terga I–II located far apart, i.e. on similar distance as on Abd. tergum III (Fig. 37). Psx formula 1/000/111101^m^. Psp formula dorsally 0/011/1111, ventrally 0/111/01^m^1^m^1^m^, coxae with 1 psp each.

Dorsal chaetotaxy, usually slightly asymmetrical, all dorsal chaetae rather long, well differentiated into macro- meso- and microchaetae, as in Fig. 37. Sensory chaetae s indistinct on body. On head p2 chaetae on the same level as p1 and p3. Chaetae p6 located anterior to pso b on head (Fig. 37). Th. tergum I with 18–21+18–21 chaetae, 1–2 chaetae m and 1–2 chaetae i present (chaetotaxy type i(1–2)2–4m(1–2)). Both Th. terga II and III with lateral microsensilla and with 5+5 or 6+6 axial microchaetae. Chaetae s' present on Abd. terga I–III and V. On Abd. tergum IV in axial area between M2 and P2 macrochaetae located 15–18 chaetae, medial chaeta p0 present (sometimes p0 absent). Abd. tergum V with one unpaired microchaeta p0 (Fig. 37). Abd. tergum VI with medial chaetae m0. Relative position of prespinal microchaetae of convergent type (Fig. 37). M/s ratio on abdominal tergum V as 18.9–26.6/15.7–20.6 (AS = 10). AS 0.8–1.1 times as long as inner edge of claw and 2.9 times longer than their basal diameter.

Chaetotaxy of ventral side of head as in Fig. 38. Perilabial area with 5(4)+5(4) a-chaetae. Postlabial chaetae 4-5+4-5 along ventral groove. Th. sterna I–III with 1–2+1–2, 2–3+2–3, 2–3+2–3 chaetae respectively. VT with ca. 11–12+11–12 chaetae, and 2–3 chaetae at base. Furcal rudiment: cuticular fold (located near middle of sternum) with 2+2 dental microchaetae in 2 rows. Chaetotaxy of manubrial field: 4 chaetae present in ma-row, 6 chaetae in mm-row, 4 chaetae in mp-row (in adult specimens) (Fig. 44). MVO absent. Each lateral anal valves with a0, 2a1 and 2a2; upper anal valve with chaetae a0, 2a2, 2b1, 2b2, c0, 2c1 and 2c2 (as in *Protaphorura jernika*, Fig. 58).

Subcoxae 1 of I, II and III legs with 7–9, 8–9, 7–8 chaetae, subcoxae 2 with 1, 5, 5, coxae with 4, 10, 12-15, trochanters with 11, 13, 13, femora with 20–21, 20–23, 20–23, tibiotarsi with four rows of chaetae (distal whorl (A+T)+B+C): 11+8+3, 11+8+4–5, 11+8+4–5 chaetae respectively. Claw with strong denticle in 1/2 of inner edge of claw and pair of lateral denticles (Figs 42, 43). Empodial appendage as long as the claw, without basal lamella. (Fig. 43).

#### Etymology.

The name of the new species refers to the Sayan Mountains in Southern Siberia, an area where the type specimens were collected.

#### Discussion.

*Protaphorura sayanica* sp. n. is probably the most similar to such Asiatic *Protaphorura* species as *Protaphorura pjasinae* (Martynova, 1976), *Protaphorura microtica* (Dunger, 1978) and *Protaphorura subarctica* (Martynova, 1976) due to the presence of the same number of pso on subcoxae 1 of all legs, ventral and dorsal side of head, Th. terga I–II and Abd. terga I–IV. However, *Protaphorura sayanica* sp. n. may easily be distinguished from these species by the number of pso on Abd. tergum V (3+3 pso in the new species and 2+2 pso all other species presented above) and presence of pair of lateral denticles on claw (absent in other four species).

### Protaphorura tuvinica sp. n.

Taxon classificationAnimaliaCollembolaOnychiuridae

http://zoobank.org/9A1C9947-6CD2-4FB3-A88C-514A0A819CD3

Figs 45–50 , 59

#### Type material.

Holotype (male): Russia, S-W Tuva, ca 30 km SW of Mugur-Aksy, upper reaches of Mugur River, Mongun-Taiga Mts, mountain tundra, moss under *Betula rotundifolia*, 2700 m alt., 50°22'N, 90°05'E, 23.VII.1993, leg. S.K. Stebaeva (SNHM). Paratypes: 10 males, 3 females and 7 juveniles, same data as holotype (SNHM – 6 paratypes: 5 males and 1 female, ISEA – 4 paratypes: 3 males and 1 female, MSPU – 4paratypes: 3 males and 1 female, and 7 juveniles).

#### Diagnosis.

PAO with 37–45 simple vesicles. Pso formula dorsally 32/022/33332, ventrally 2/000/0001, subcoxae 1 of I–III legs without pso. Submedial pso a and b on Abd. terga I–II located far apart. Psx formula on Abd. sterna: 110–1001 ^m^. Th. tergum I with 9–11+9–11 chaetae, chaeta m absent. Chaetae s' absent on abdominal terga I–III and V. Manubrial field with 19 chaetae in 4 rows. Claw without lateral denticles.

#### Description.

**Figures 45–50.**
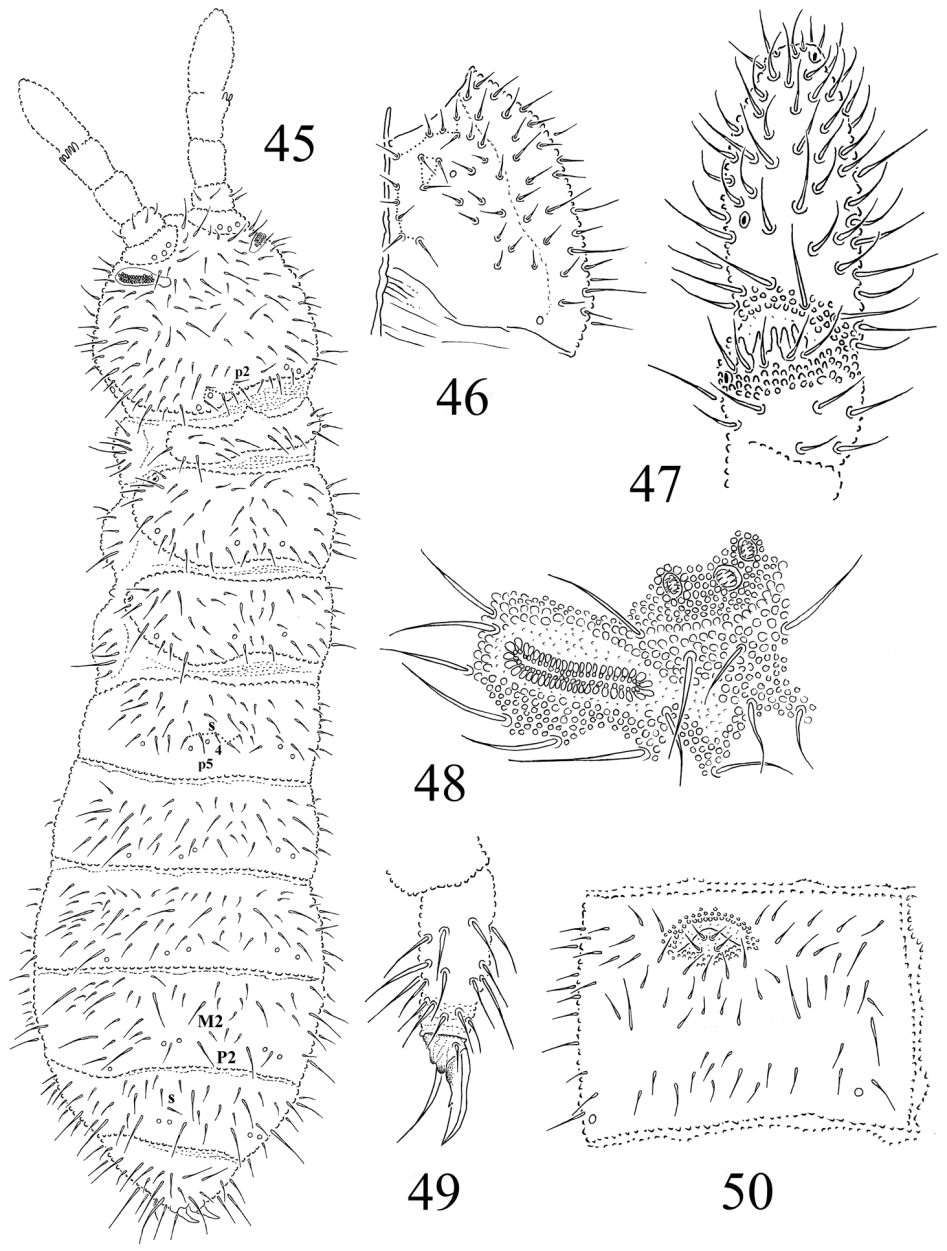
*Protaphorura tuvinica*: **45** habitus and dorsal chaetotaxy **46** right part of head ventrally **47** dorsal side of Ant. IIIIV **48** PAO and anterior cephalic pseudocelli **49** tibiotarsal chaetotaxy and claw of leg III **50** chaetotaxy of Abd. sternum IV.

Holotype (male) length 1.9 mm, length of paratypes: 1.7–1.8 mm (males) and 1.9–2.2 mm (females). Shape of body typical for the genus: cylindrical with strong AS on distinct papillae (Fig. 45). Colour in alcohol yellowish-white. Granulation more or less uniform, distinct. Usually 11–12 grains around each pso.

Antennae slightly shorter than head, their base well marked. Ant. I with 9–10 chaetae, Ant. II with 17 chaetae. AIIIO consisting of 5 guard chaetae, 5 papillae, 2 smooth sensory rods, 2 straight and granulated sensory clubs, ventro-lateral microsensillum present (Fig. 47). Ant. IV with subapical organite in unprotected cavity without clear cuticular papilla. Microsensillum on Ant. IV in usual position above second proximal row of chaetae. Ventrally Ant. IV with numerous chaetae (ca. 68–70). Sensilla indistinct on Ant. IV (Fig. 47).

PAO of middle length, consisting of 37–45 simple vesicles (Fig. 48). Labral formula of chaetae: 4/342. Maxillary outer lobe with simple palp, basal chaeta and with two sublobal hairs. Labial palp of type A. Labium with 7 proximal, 4 basomedian (E, F, G, and f), and 6 basolateral chaetae (a, b, c, d, e, e’). Papillae A-E with 1, 4, 0, 3, 3 guard chaetae respectively.

Pso formula dorsally 32/022/33332, ventrally 2/000/0001 (Figs 45, 46, 50). Subcoxae 1 of I–III legs without pso and with one psx each. Submedial pso a and b on Abd. terga I–II located rather far apart, i.e. on similar distance as on Abd. tergum III (Fig. 45). Psx formula 0/000/110(1)01. Psp formula dorsally 0/011/1111, ventrally 0/111/01^m^1^m^1^m^, coxae with 1 psp each.

Dorsal chaetotaxy rather symmetrical and plurichaetotic, chaetae well differentiated into macrochaetae and microchaetae (fig. 45). Sensory chaetae s indistinct on body. On head p2 chaetae displaced forward in relation to p1 and p3. Chaetae p6 on head located between pso a and b (Fig. 45). Th. tergum I with 9–11+9–11 chaetae, chaeta m absent (chaetotaxy type i2-). Both Th. terga II and III with lateral microsensilla and with 5+5 or 6+6 axial microchaetae. Chaetae s' absent on Abd. terga I–III and V. On Abd. tergum IV in axial area between M2 and P2 macrochaetae located 7–8 chaetae, medial chaeta m0 present (Fig. 45). Abd. tergum V usually with 1–2 unpaired microchaeta m0 and p0 (often m0 absent) (Fig. 45). Abd. tergum VI with medial chaetae m0. Relative position of prespinal microchaetae of parallel type (Fig. 45). M/s ratio on Abd. tergum V as 14.6–17.2/4.6–6.2 (AS = 10). AS 0.9–1.0 as long as inner edge of claw and 3.1 times longer than their basal diameter.

Chaetotaxy of ventral side of head as in Fig. 46. Perilabial area with 5+5 a-chaetae (Fig. 46). Postlabial chaetae 5-6+5-6 along ventral groove. Th. sterna I–III without chaetae. VT with ca. 8–9+8–9 chaetae and 2 chaetae at base. Furcal rudiment: cuticular fold (located on the anterior edge of the sternum) with 2+2 dental microchaetae in 2 rows. Chaetotaxy of manubrial field: 5 chaetae present in ma-row, 4 chaetae in mm’-row, 6 chaetae in mm-row and 4 chaetae in mp-row (Fig. 50). MVO absent. Each lateral anal valves with a0 and 2a1 (a2 absent); upper anal valve with chaetae a0, 2a2, 2b1, 2b2, c0, 2c1 and 2c2 (as in *Protaphorura vasilinae*, Fig. 59).

Subcoxae 1 of I, II and III legs with 5–6, 6, 5 chaetae, subcoxae 2 with 1, 5, 5, coxae with 3, 10, 13, trochanters with 11, 12, 10, femora with 20, 20, 19–20, tibiotarsi with four rows of chaetae (distal whorl (A+T)+B+C): 11+8+3, 11+8+3, 11+8+3-4 chaetae respectively. Claw with strong denticle in 1/2 of inner edge of claw (Fig. 49). Empodial appendage of the same length as inner edge of claw, without basal lamella. (Fig. 49).

#### Etymology.

The name of the new species refers to the Tuva Republic (Russian Federation), the place where the type specimens were collected.

#### Discussion.

*Protaphorura tuvinica* sp. n. belongs to the group of *Protaphorura* species without pseudocelli on subcoxa 1 of all legs and with 2+2 pso ventrally on head: *Protaphorura ombrophila* (Stach, 1960), *Protaphorura kopetdagi* Pomorski, 1994, *Protaphorura salsa* Kaprus’, Paśnik & Weiner, 2014, *Protaphorura bakhchisaraica* Kaprus’, Paśnik & Weiner, 2014 and *Protaphorura ajudagi* Pomorski, Skarżyński & Kaprus’, 1998. All these species inhabit the territory of southern Palearctic from Crimean Peninsula to central Asia and southern Siberia.

The new species has the pseudocellar formula the same as in *kopetdagi* (32/022/33332) when the other posses the different number of pseudocelli. The males of *Protaphorura kopetdagi*, *Protaphorura salsa*, *Protaphorura bakhchisaraica* and *Protaphorura ajudagi* are armed with the male ventral organ whereas the new species and *Protaphorura ombrophila* have males devoided of the organ. *Protaphorura tuvinica* differs also from the latter species by the number of pso on Abd. terga IV-V (3,2 in the new species and 4,2 in *Protaphorura ombrophila*).

### Protaphorura vasilinae sp. n.

Taxon classificationAnimaliaCollembolaOnychiuridae

http://zoobank.org/80C4CF4F-0711-488A-AB00-5EEFAEA30B20

Figs 51–57 , 59

#### Type material.

Holotype (female): Russia, West Siberia, 25 km S of Novosibirsk, Akademgorodok, lawn, soil, 400 m alt., 54°49'N, 83°08'E, 2.X.1994, leg. S.K. Stebaeva (SNHM). Paratypes: 7 females and 6 juveniles, same data as holotype (SNHM – 3 paratype females and 3 juveniles, ISEA – 4 paratype females and 3 juveniles); 2 females and 3 juveniles: Russia, N-E Altai, Turochak Region, meadow, soil, 11.VI.2002, leg. E. Sleptsova (SNHM).

#### Diagnosis.

PAO with 32–36 simple vesicles. Pso formula dorsally 32/022/33332, ventrally 2/000/0001, subcoxae 1 of I–III legs with 1,1,1 pso respectively. Submedial pso a and b on Abd. terga I–II located far apart. Psx formula on Abd. sterna: 110001^m^. Th. tergum I with 10–11+10– 11 chaetae, chaeta m absent. Chaetae s' absent on Abd. terga I–III and present on Abd. tergum V. Manubrial field with 25–28 chaetae in 6 rows. Claw without lateral denticles.

#### Description.

**Figures 51–57.**
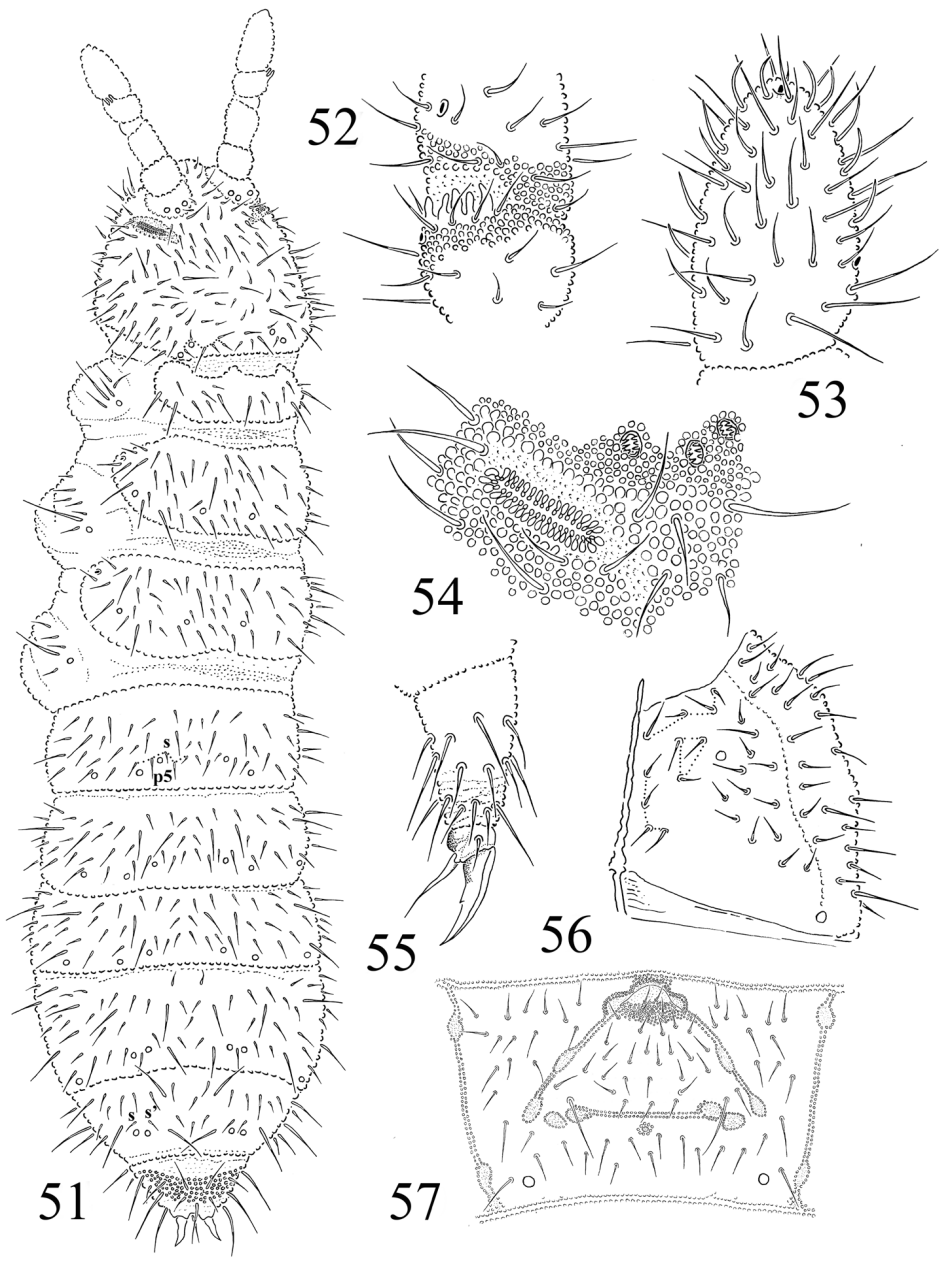
*Protaphorura vasilinae*: **51** habitus and dorsal chaetotaxy **52** AIIIO **53** ventral side of Ant. IV **54** PAO and anterior cephalic pseudocelli **55** tibiotarsal chaetotaxy and claw of leg III **56** right part of head ventrally **57** chaetotaxy of Abd. sternum IV.

Holotype (female) length 1.5 mm, length of paratypes: 1.4–1.7 mm (females). Shape of body typical of the genus: cylindrical with strong AS on distinct papillae (Fig. 51). Colour in alcohol yellowish-white. Granulation more or less uniform, distinct. Usually 10–12 grains around each pso.

Antennae slightly shorter than the head, their base well marked. Ant. I with 10 chaetae, Ant. II with 16 chaetae. AIIIO consisting of 5 guard chaetae, 5 papillae, 2 smooth sensory rods, 2 straight and granulated sensory clubs, ventro-lateral microsensillum present (Fig. 52). Ant. IV with subapical organite in unprotected cavity without clear cuticular papilla (Fig. 53). Microsensillum on antennal segment IV in usual position above second proximal row of chaetae. Ventrally Ant. IV with numerous chaetae (ca. 50–55). Ant. IV with 8–11 slightly differentiated sensilla (Fig. 53).

PAO of middle length, consisting of 32–36 simple vesicles (Fig. 54). Labral formula of chaetae: 4/342. Maxillary outer lobe with simple palp, basal chaeta and with two sublobal hairs. Labial palp of type A. Labium with 7 proximal, 4 basomedian (E, F, G, and f), and 6 basolateral chaetae (a, b, c, d, e, e’). Papillae A-E with 1, 4, 0, 3, 3 guard chaetae respectively.

Pso formula dorsally 32/022/33332, ventrally 2/000/0001 (Figs 51, 56, 57). Subcoxae 1 of I– III legs with one pso and one psx each. Submedial pso a and b on Abd. terga I–II located far apart, i.e. on similar distance as on Abd. tergum III (Fig. 51). Psx present on Abd. sterna I–II and VI (psx formula 0/000/110001^m^). Psp formula dorsally 0/011/1111, ventrally: 0/111/01^m^1^m^1^m^ , coxae with 1 psp each.

Dorsal chaetotaxy rather symmetrical, as in Fig. 51. Dorsal chaetae well differentiated into macrochaetae and microchaetae. Sensory chaetae s indistinct on body. On head p2 chaetae are displaced forward in relation to p1 and p3. Chaetae p6 located between pseudocelli a and b on head. Th. tergum I with 10–11+10–11 chaetae, chaeta m absent (chaetotaxy type i2–3-). Both Th. terga II and III with lateral microsensilla and with 5+5 or 6+6 axial microchaetae. Chaetae s' absent on Abd. terga I–III and present on Abd. tergum V. On Abd. tergum IV in axial area between M2 and P2 macrochaetae located 7–8 chaetae, medial chaeta m0 present (Fig. 51). Abd. tergum V usually with 2 unpaired microchaeta m0 and p0 (sometimes m0 absent) (Fig. 51). Abd. tergum VI with medial chaetae m0. Relative position of prespinal microchaetae of subparallel type (Fig. 51). M/s ratio on abdominal tergum V as 14.9–16/5.6–5.2 (AS = 10). AS 1.1 times longer than inner edge of claw and 3.1 times longer than their basal diameter.

Chaetotaxy of ventral side of head as in Fig. 56. Perilabial area with 4+4 a-chaetae. Postlabial chaetae 4–5+4–5 along ventral groove. Th. sterna I–III without chaetae. VT with ca. 8–9+8–9 chaetae, and 2 chaetae at base. Chaetotaxy of Abd. sternum IV as in Fig. 57. Furcal rudiment: cuticular fold (located on the anterior edge of the sternum) with 2+2 dental microchaetae in 2 rows. Chaetotaxy of manubrial field: 4 chaetae present in ma-row, 4 chaetae in ma’-row, 4–5 chaetae in mm’’-row, 5–6 chaetae in mm’-row, 4 chaetae in mm-row and 4–5 chaetae in mp-row (in adult specimens) (Fig. 56). MVO absent. Each lateral anal valves with a0 and 2a1 (a2 absent); upper anal valve with chaetae a0, 2a2, 2b1, 2b2, c0, 2c1 and 2c2 (Fig. 59).

**Figures 58–59.**
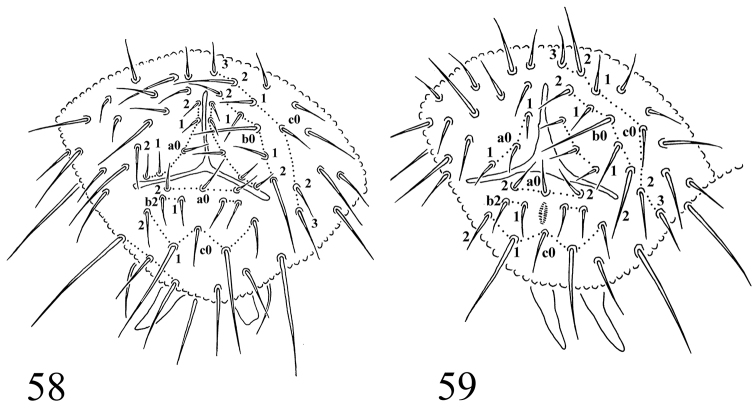
Chaetotaxy of anal valves: **58** *Protaphorura jernika* **59** *Protaphorura vasilinae*.

Subcoxae 1 of I, II and III legs with 5, 6–7, 6 chaetae, subcoxae 2 with 1, 5, 4, coxae with 3, 10–11, 13, trochanters with 11, 11, 10, femora with 18, 18, 18, tibiotarsi with four rows of chaetae (distal whorl (A+T)+B+C): 11+8+3, 11+8+3, 11+8+4 chaetae respectively. Claw with strong denticle in the 1/2 of inner edge of claw (Fig. 55). Empodial appendage of the same length as inner edge of claw, without basal lamella. (Fig. 55).

#### Etymology.

The species is cordially dedicated to Vasilina, a granddaughter of Dr. Sophya Stebaeva.

#### Discussion.

The same number of pso on body ventrally (2+2 on head and 1+1 on Abd. sternum V), the presence of pseudocelli on subcoxae 1 of all legs, 2+2 posterior cephalic pso and 2+2 pso on Abd. tergum V allow suggesting a close similarity between *Protaphorura vasilinae* sp. n. and *Protaphorura jernika* sp. n. (see also discussion of *Protaphorura jernika* sp. n.). These species distinctly differ in the number of pso on Th. terga II–III and Abd. tergum IV (2+2,2+2 and 3+3 in *Protaphorura vasilinae* vs 3+3,3+3 and 4+4 in *Protaphorura jernica* respectively), in the formula of psx on Abd. sterna (110001m in *Protaphorura vasilinae* vs 111000 in *Protaphorura jernica*) and in the chaetotaxy of Th. tergum I (chaetotaxy type i2–3- in *Protaphorura vasilinae* vs i2–3m in *Protaphorura jernica*) and Abd. tergum V (s’ present in *Protaphorura vasilinae* vs s’ absent in *Protaphorura jernica*).

### Key to *Protaphorura* species of the Eastern Palearctic

For the species with high variability in the pseudocellar formula we used in the key the most common type.

**Table:** 

1	AIIIO with four papillae	**2**
–	AIIIO with five papillae	**5**
2	Th. terga II and III with 3+3 pso each (formula of dorsal pso: 33/033/33333)	***Protaphorura matsumotoi* (Kinoshita, 1923)** (Japan)
–	Th. terga II and III with 1+1 and 2+2 pso respectively	**3**
3	Ventrally on head 1+1 pso present in posterolateral position, formula of dorsal pso: 33/012/33342	***Protaphorura dzherga* Gulgenova & Potapov, 2013** (Russia: Transbaikal)
–	Ventral pso on head absent	**4**
4	Formula of dorsal pso: 33/012/33332, furcal area with two pairs of dental microchaetae	***Protaphorura dorzhievi* Gulgenova & Potapov, 2013** (Russia: Transbaikal)
–	Formula of dorsal pso:43/012/33353, furcal area with one pair of dental microchaetae	***Protaphorura uniparis* Gulgenova & Potapov, 2013** (Russia: Transbaikal)
5	AIIIO with two slender, long sensory rods: one inserted dorsal to the papillae, secod between papillae	**6**
–	AIIIO with two normal sensory rods inserted behind the papillae , between the sensory cluba	**8**
6	Antennal base with four pso (formula of dorsal pso: 43(4)/022/3(4)3(4)3(4)5(6)3(4)), PAO with 4246 vesicles	***Protaphorura nutak* (Yosii, 1972)** (Russia: Far East, Kunashir Island and Eastern Siberia, Japan)
–	Antennal base with three pso	**7**
7	Formula of dorsal pso: 33/022/33343, PAO with about 45 vesicles	***Protaphorura longisensillata* (Yosii, 1969)** (Japan)
–	Formula of dorsal pso: 32/022/33342, PAO with 3640 vesicles	*Protaphorura diplosensillata* (Dunger, 1978)* (Mongolia)
8	Subcoxae1 of legs I, II and III without pso	**9**
–	Subcoxae1 of legs I, II and III with 1,0,0 pso respectively	**21**
–	Subcoxae1 of legs I, II and III with 1,1,1 pso respectively	**27**
9	Ventral pso on head absent	**10**
–	Ventrally on head 1+1 or 2+2 pso present	**11**
10	Formula of dorsal pso: 32/022/33333, PAO with 4065 vesicles, MVO absent	***Protaphorura borealis* (Martynova, 1973 in Martynova, Gorodkov & Chelnokov, 1973)** (Eastern Palearctic)
–	Formula of dorsal pso: 33/012/33332, PAO with 2126 vesicles, MVO in a form of two brush-shape chaetae on each anal valve	***Protaphorura minima* Sun, Zhang & Wu, 2013** (North Eastern China)
11	Ventrally on head 2+2 pso present, Abd. sternum IV with 1+1 pso	**12**
–	Ventrally on head 1+1 pso present in anteromedial position, Abd. sternum IV without pso	**15**
12	Abd. tergum V with 3+3 pso	**13**
–	Abd. tergum V with 2+2 pso	**14**
13	Anterolateral pso on Abd. tergum IV present, formula of dorsal pso: 32(3)/022(3)/33(2)3(2)43, MVO absent	***Protaphorura ombrophila* (Stach, 1960)** (Afghanistan)
–	Anterolateral pso on Abd. tergum IV absent, formula of dorsal pso: 33/022/3324(3)3, MVO present on Abd. sterna IIIII with 2+2 and 1+1 modified chaetae respectively	***Protaphorura salsa* Kaprus’, Paśnik & Weiner, 2014** (Russia: southern Siberia)
14	PAO with 3745 vesicles, formula of dorsal pso: 32/022/33332, MVO absent	***Protaphorura tuvinica* sp.n.** (Russia: southern Siberia)
–	PAO with 2636 vesicles, formula of dorsal pso: 32/022/33332, MVO present on Abd. sterna IIIII with 2+2 and 2+2 modified chaetae respectively	***Protaphorura kopetdagi* Pomorski, 1994** (Turkmenistan: Kopetdag Mts.)
15	Antennal base with four or more pso	**16**
–	Antennal base with three pso	**17**
16	Dorsomedial pso on Th. tergum II and anterolateral pso on Abd. tergum IV present (formula of dorsal pso: 4(5,6)3(4)/022/3335(4)3(4,5))	***Protaphorura octopunctata* (Tullberg, 1876)** (Russia: central Siberia)
–	Dorsomedial pso on Th. tergum II and anterolateral pso on Abd. tergum IV absent (formula of dorsal pso: 43/012/333(2)43)	***Protaphorura tolae* Pomorski & Kaprus’, 2007** (Russia: eastern Siberia)
17	Posterior cephalic pso 2+2, claws with pair lateral denticles	**18**
–	Posterior cephalic pso 3+3, claws without lateral denticles	**20**
18	Th. tergum I in adult specimens with 11+11 chaetae, claws with strong lateral denticles, formula of dorsal pso: 32/022/33232	***Protaphorura microcellata* (Dunger, 1978)** (Mongolia)
–	Th. tergum I in adult specimens with 1725+1725 chaetae	**19**
19	Th. tergum I with 1720+1720 chaetae, formula of dorsal pso: 32/022/33332, claws with very small lateral denticles	**Protaphorura cf. microcellata (Dunger, 1978)** (Russia: central Siberia after Babenko & Kaprus’, 2014)
–	Th. tergum I with 2325+2325 chaetae, formula of dorsal pso: 32/011/22232, claws with strong lateral denticles	***oligopseudocellata* sp. n.** (Russia: southern Siberia)
20	Formula of dorsal pso: 33/022/33332, ventral psx formula: 01/000/111100, chaetae s’ present on Abd. terga IIII and V	***Protaphorura bicampata* (Gisin, 1956)** (Northern Europe, Eastern Palearctic)
–	Formula of dorsal pso: 33/01(2)2/3334(3)2, ventral psx formula: 01/000/100000, chaetae s’ absent on Abd. terga IIII and V	***Protaphorura jacutica* (Martynova, 1976)** (north eastern Europe, eastern Asia)
21	Antennal base with four pso	**22**
–	Antennal base with three pso	**24**
22	Abd. tergum IV with 5+5 pso (formula of dorsal pso: 43/022/33353), PAO with 4042 vesicles	***Protaphorura maoerensis* Sun, Wu & Gao, 2013** (noth eastern China)
–	Abd. tergum IV with 4+4 pso	**23**
23	Formula of dorsal pso: 43/022/33342, claws always with strong inner denticle, PAO with 2627 vesicles	***Protaphorura mongolica* (Martynova, 1975)** (Mongolia)
–	Formula of dorsal pso: 43/022/33343, claws without or rarely with very small inner denticle (in Asian populations), PAO with 3035 vesicles	***Protaphorura sakatoi* (Yosii, 1966)** (central and south-eastern Europe, Russia: Caucasus Mts and southern Siberia, Afghanistan, Kazakhstan, Tajikistan)
24	Abd. sternum IV with 1+1 pso, formula of dorsal pso: 32/012/33132	***Protaphorura brevispinata*** (southern Korea)
–	Abd. sternum IV without pso	**25**
25	Posterior cephalic pso 2+2 (formula of dorsal pso: 32/012/33232), psx formula on Abd. sterna IVI: 100000	***Protaphorura changbaiensis* Sun, Zhang & Wu, 2013** (north eastern China)
–	Posterior cephalic pso 3+3 (formula of dorsal pso: 33/022/33342)	**26**
26	Claws without inner denticle, chaeta a0 present on Abd. tergum VI, prespinal chaetae placed convergently	***Protaphorura zori* (Martynova, 1975 in Martynova & Chelnokov, 1975)** (Tajikistan: eastern Pamir)
–	Claws with strong inner denticle, chaeta a0 absent on Abd. tergum VI, prespinal chaetae placed divergently	***Protaphorura nikolai* sp. n.** (Russia: Far East)
27	Ventrally on head 2+2 pso present	**28**
–	Ventrally on head 1+1 pso present in anteromedial position	**31**
28	Abd. sternum IV without pso	**29**
–	Abd. sternum IV with 1+1 pso	**30**
29	Formula of dorsal pso: 32(3)/012/33342, claws with inner denticle, PAO with 1213 vesicles	***Protaphorura buryatica* Gulgenova & Potapov, 2013** (Russia: Transbaikal)
–	Formula of dorsal pso: 43/02(3)2(3)/3335(4,6)3(4), claws without inner denticle, PAO with 1622 vesicles	***Protaphorura merita* Kaprus’ & Pomorski, 2008** (Russia: southern Siberia)
30	Formula of dorsal pso: 32/033/33342, psx formula on Abd. sterna IVI: 111000	***Protaphorura jernika* sp. n.** (Russia: southern Siberia)
–	Formula of dorsal pso: 32/022/33332, psx formula on Abd. sterna IVI: 110001^m^	***Protaphorura vasilinae* sp. n.** (Russia: southern Siberia)
31	Antennal base with four or more pso	**32**
–	Antennal base with three pso	**36**
32	Th. tergum III with 2+2 pso (formula of dorsal pso: 43/022/33342), psx formula 1/000/110001^m^	***Protaphorura licheniphila* Kaprus’ & Pomorski, 2008** (Russia: central Siberia)
–	Th. tergum III with 3+3 pso	**33**
33	Abd. tergum V with 2+2 pso (formula of dorsal pso: 43/023/33342), PAO with 1822 vesicles	***Protaphorura nazarovensis* Kaprus’ & Pomorski, 2008** (Russia: south Siberia)
–	Abd. tergum V with 3+3 or more pso	**34**
34	Abd.terga I–III and V without chaetae s’, formula of dorsal pso: 43/023/33353, PAO with 1622 vesicles, psx formula on Abd. sterna IVI: 111101^m^	***Protaphorura jiamusiensis* Sun, Wu & Gao, 2013** (north eastern China)
–	Abd.terga I–III and V with chaetae s’	35
35	PAO with 1826 vesicles, psx formula on Abd. sterna IVI: 111101^m^, formula of dorsal pso: 4(5)3(4,5)/033/4(3)4(3)4(3)5(6)3(4)	***Protaphorura submersa* Kaprus’ & Pomorski, 2008** (Russia: southern Siberia)
–	PAO with 3640 vesicles, psx formula on Abd. sterna IVI: 100001?^m^ , formula of dorsal pso: 4(5,6)4/03(2)3(2)/4(3)4(3)4(3,5)5(6)4(3)	***Protaphorura elenae* Kaprus’ & Pomorski, 2008** (Russia: eastern Siberia)
36	Posterior cephalic pso 2+2	**37**
–	Posterior cephalic pso 3+3	**42**
37	Th. terga II and III with 3+3 pso each (formula of dorsal pso: 32/033/33343)	***Protaphorura abscondita* sp. n.** (Russia: southern Siberia)
–	Th. Terga II and III with 2+2 pso	**38**
38	Abd. terga IIII without chaetae s’	**39**
–	Abd. terga IIII with chaetae s’	**40**
39	Subapical organite on Ant. IV in cavity protected by cuticular papillae, PAO with 30–42 simple vesicles, most common formula of dorsal pso: 32/022/33343, but some specimens may have 3+3 posterior pso on head and 2+2 pso on Abd. tergum V	***Protaphorura tschernovi* (Martynova, 1976)** (Russia: western Taimyr, central Siberia)
–	Subapical organite on Ant. IV in unprotected cavity, PAO with 25–40 simple vesicles, formula of dorsal pso: 32/022/3333(4)2	***Protaphorura subarctica* (Martynova, 1976)** (Northern Palearctic)
40	Abd. tergum V with chaetae s’ PAO with about 41–48 vesicles, formula of dorsal pso: 32/022/33343 and ventral pso: 1/000/0000	***Protaphorura sayanica* sp. n.** (Russia: southern Siberia)
–	Abd. tergum V without chaetae s’	**41**
41	Submedial pso a and b on Abd. terga I-II in nearby position and both these pso set medially to macrochaetae p5, formula of dorsal pso: 32/022/3334(3)2, PAO with 24‒40 simple vesicles	***Protaphorura pjasinae* (Martynova, 1976)** (northern Asia, western Siberia)
–	Submedial pso a and b on Abd. terga I-II set far apart and pso b set laterally to macrochaetae p5, formula of dorsal pso: 32/022/33342, PAO with 22 simple vesicles	***Protaphorura microtica* (Dunger, 1978)** (Mongolia)
42	Th. tergum II with 1+1 pso (formula of dorsal pso: 33/012/33342), psx formula on Abd. sterna IVI: 111101^m^, PAO with 2432 vesicles	***Protaphorura genheensis* Sun, Chang & Wu, 2015** (north eastern China)
–	Th. tergum II with 2+2 or more pso	**43**
43	Abd. tergum IV with 3 +3 pso (formula of dorsal pso: 33/022/33333), claws without inner denticle	***Protaphorura fimata* (Gisin, 1952)** (Kyrgyzstan, Iran)
–	Abd. tergum IV with 4 +4 or more pso	**44**
44	Chaetae s’ present on Abd. terga IIII or V	**45**
–	Chaetae s’ absent on Abd. terga IIII or V	**48**
45	AS less than 0.5 length of claws III, formula of dorsal pso: 33/022/33343	***Protaphorura ussurica* (Martynova, 1981)** (Russia: Far East)
–	AS 0.7–1.0 length of claws III	**46**
46	Relative position of prespinal microchaetae on Abd. tergumVI parallel type, formula of dorsal pso: 33(2)/022/33342(3), psx formula on Abd. sterna IVI: 110001^m^	***Protaphorura boedvarssoni* Pomorski, 1993** (Russia: western and central Siberia)
–	Relative position of prespinal microchaetae on Abd. tergumVI distinctly convergent type	**47**
47	Formula of dorsal pso highly variable: 33(2)/03(2)3(2)/4(3,5)4(3,5)4(3,5,6)5(4,6)3(2,4), chaetae s on Abd. tergum V 1.01.1 times longer than AS	***Protaphorura tundricola* (Martynova, 1976)** (north eastern Europe, western and central Siberia)
–	Formula of dorsal pso: 33/022(3)/3334(5,6)2(3), chaetae s on Abd. tergum V 1.5 times longer than AS	***Protaphorura neriensis* (Martynova, 1976)** (Russia: eastern Siberia)
48	Th. tergum II with 3+3 pso (formula of dorsal pso: 3(4)3/033/33342), claws with hardly noticeable inner denticle	***Protaphorura kaszabi* (Dunger, 1978)** (Mongolia, north eastern China)
–	Th. tergum II with 2+2 or rarely 1+1 pso (formula of dorsal pso: 33/02(1)2(3)/33342(3), claws with clear inner denticle	***Protaphorura taimyrica* (Martynova, 1976)** (northern Asia)

### Species insufficient described which are not included in the key

*Protaphorura aksuensis* (Martynova, 1972), formula of dorsal pso: 33/022/33333, (Kyrgyzstan)

*Protaphorura tridentata* (Stebaeva, 1982), formula of dorsal pso: 32/022/33342, (southern Siberia)

*Protaphorura teres* (Yosii, 1956), formula of dorsal pso: 32/022/33333, (Japan)

*Protaphorura yagii* (Miyoshi, 1923), formula of dorsal pso: 32/022/33232, (Japan)

## Supplementary Material

XML Treatment for Protaphorura abscondita

XML Treatment for Protaphorura jernika

XML Treatment for Protaphorura nikolai

XML Treatment for Protaphorura oligopseudocellata

XML Treatment for Protaphorura ombrophila

XML Treatment for Protaphorura sayanica

XML Treatment for Protaphorura tuvinica

XML Treatment for Protaphorura vasilinae
